# ﻿Morphological and phylogenetic analyses reveal three new species of *Fusarium* (Hypocreales, Nectriaceae) associated with leaf blight on *Cunninghamialanceolata* in China

**DOI:** 10.3897/mycokeys.101.113128

**Published:** 2024-01-08

**Authors:** Jiao He, De-Wei Li, Wen-Li Cui, Li-Hua Zhu, Lin Huang

**Affiliations:** 1 Co-Innovation Center for Sustainable Forestry in Southern China, Nanjing Forestry University, Nanjing, Jiangsu 210037, China Nanjing Forestry University Nanjing China; 2 The Connecticut Agricultural Experiment Station Valley Laboratory, Windsor, CT 06095, USA The Connecticut Agricultural Experiment Station Windsor United States of America

**Keywords:** *Cunninghamialanceolata*, *Fusarium*, leaf blight, new species, pathogenicity

## Abstract

Chinese fir (*Cunninghamialanceolata*) is a special fast-growing commercial tree species in China with high economic value. In recent years, leaf blight disease on *C.lanceolata* has been observed frequently. The diversity of *Fusarium* species associated with leaf blight on *C.lanceolata* in China (Fujian, Guangxi, Guizhou, and Hunan provinces) was evaluated using morphological study and molecular multi-locus analyses based on RNA polymerase second largest subunit (*RPB2*), translation elongation factor 1-alpha (*TEF-1α*), and RNA polymerase largest subunit (*RPB1*) genes/region as well as the pairwise homoplasy index tests. A total of five *Fusarium* species belonging to four *Fusarium* species complexes were recognized in this study. Two known species including *Fusariumconcentricum* and *F.fujikuroi* belonged to the *F.fujikuroi* species complex, and three new *Fusarium* species were described, i.e., *F.fujianense* belonged to the *F.lateritium* species complex, *F.guizhouense* belonged to the *F.sambucinum* species complex, and *F.hunanense* belonged to the *F.solani* species complex. To prove Koch’s postulates, pathogenicity tests on *C.lanceolata* revealed a wide variation in pathogenicity and aggressiveness among the species, of which *F.hunanense* HN33-8-2 caused the most severe symptoms and *F.fujianense* LC14 led to the least severe symptoms. To our knowledge, this study also represented the first report of *F.concentricum*, *F.fujianense*, *F.fujikuroi*, *F.guizhouense*, and *F.hunanense* causing leaf blight on *C.lanceolata* in China.

## ﻿Introduction

The genus *Fusarium* (Nectriaceae) is one of the most renowned genera that contains many phytopathogenic fungi. The members of this genus can directly incite diseases in plants, humans, and domesticated animals (Rabodonirina et al. 1994; Boonpasart et al. 2002; Vismer et al. 2002). *Fusarium* was included in the top 10 globally most important genera of plant pathogenic fungi based on scientific and economic importance (Dean et al. 2012), in particular because of the members of the *F.sambucinum* species complex (FSAMSC) and *F.oxysporum* species complex (FOSC) (O’Donnell et al. 2015; Gräfenhan et al. 2016) that comprises some of the most destructive agricultural pathogens. *Fusariumgraminearum* and 21 related species comprising the *F.sambucinum* species complex lineage 1 (FSAMSC-1) are the most important *Fusarium* head blight (FHB) pathogens of cereal crops world-wide (Goswami and Kistler 2005; Kelly et al. 2016). Further impactful fusaria include the members of the *F.fujikuroi* species complex (FFSC), *F.verticillioides* (teleomorphic synonym, *Gibberellamoniliformis*), *F.fujikuroi* (teleomorphic synonym, *G.fujikuroi*), and *F.proliferatum* (teleomorphic synonym, *G.intermedia*), which are well known for their abilities to cause devastating diseases, such as rice bakanae, maize ear rot and soybean root rot, leading to considerable reductions in crop yields and economic income (O’Donnell et al. 2015; Qiu et al. 2020). The members of the *F.solani* species complex (FSSC) cause plant diseases, mostly root and crown rots and vascular wilts on a wide range of plants, including soybeans, potato, cucurbits, peas, sweet potato, Chinese rose, and various legumes (Coleman 2016; Summerell 2019; He et al. 2021).

There has been confusion in *Fusarium* taxonomy for a long time because of the nine-species system of Snyder and Hansen (1940), the misleading overlaps caused by convergent evolution and character loss, the phenomenon of cultural degeneration, and firm opinions of the taxonomists and plant pathologists who have been working on them. First described by Link (1809) and typified by *Fusariumroseum* (presently *F.sambucinum* nom. cons.) (Gams et al. 1997), the generic and species concepts in *Fusarium* have endured significant changes since the cornerstone of phenotypically-based taxonomic treatments that grouped species into sections, morphological varieties or forms and later formae speciales based on pathogenicity and host ranges (Wollenweber and Reinking 1935; Snyder and Hansen 1940; Toussoun and Nelson 1968; Gerlach and Nirenberg 1982; Nelson et al. 1983; Burgess et al. 1988). Later, the species were redistributed into species complexes after the introduction of modern molecular tools (O’Donnell et al. 2000; Geiser et al. 2013; O’Donnell et al. 2013; Aoki et al. 2014). O’Donnell et al. (2022) indicates that *Fusarium* is assessed to have >400 phylospecies and *ca.* 1/3 of the phylospecies have not been formally described; clearly, morphology alone is insufficient to differentiate most of these species. To solve the species delimitation and identification dilemma, a polyphasic approach has gradually been applied and several online databases (*Fusarium*-ID, *Fusarium* MLST and FUSARIOID-ID) have been established based on different taxonomic opinions (O’Donnell et al. 2012; Crous et al. 2021; Torres-Cruz et al. 2022). Despite these significant contributions, debates surrounding the generic delimitation of *Fusarium* and whether the genus *Neocosmospora* (also known as *F.solani* species complex, FSSC) belongs to *Fusarium* remain (Crous et al. 2021; Geiser et al. 2021; Wang et al. 2022). There has been a consensus for over a century that the FSSC is part of *Fusarium*, which was affirmed by molecular phylogenetic analyses and codified in a proposal to recognize *Fusarium* as a monophyletic group that includes the FSSC (Geiser et al. 2013). A disagreement on the generic concept of *Fusarium* has become more contentious in the last decade. Geiser et al. (2013) advocated “recognizing the genus *Fusarium* as the sole name for a group that includes virtually all *Fusarium* species of importance in plant pathology, mycotoxicology, medicine, and basic research”, and the retained genus *Fusarium* includes *F.solani* species complex (FSSC). This treatment was subsequently challenged by Lombard et al. (2015) who split the genus *Fusarium* into seven genera and segregated the FSSC as *Neocospmospora*. Later, Sandoval-Denis and Crous (2018) and Sandoval-Denis et al. (2019) justified the treatment of Lombard et al. (2015) based on the phylogenetic analyses using four loci and dispute that the Geiser et al. (2013) concept of *Fusarium* is polyphyletic. O’Donnell et al. (2020) rebutted the polyphyletic conclusions of Sandoval-Denis and Crous (2018) and Sandoval-Denis et al. (2019). Geiser et al. (2021) examined the conclusion of Sandoval-Denis and Crous (2018) and Sandoval-Denis et al. (2019), developed a phylogeny according to sequences of 19 orthologous protein-coding genes and show that *Fusarium* including the FSSC is monophyletic. Thus, 40 species described as *Neocosmospora* are recently recombined in *Fusarium* (Aoki et al. 2020, 2021a, b). Crous et al. (2021) insist that fusarium-like are polyphyletic in Nectriaceae and dispute that a narrower generic concept with a combination of features is necessary for the majority of fusarioid species based on the phylogenetic analyses using sequence data of eight loci. They segregate the Wollenweber concept of *Fusarium* into 20 genera with synapomorphic characteristics (Crous et al. 2021). O’Donnell et al. (2022) opined that *Fusarium* remains the best scientific, nomenclatural and practical taxonomic option available. However, the disagreement is far from settled.

The narrow generic concept of *Fusarium* is leading to a large number of name changes and confusions among plant pathologists, medical mycologists, quarantine officials, regulatory agencies, biologists, and other professionals. Rebuilding the correct systematic position of a large number of fungal names cannot be achieved without repeated studies (de Hoog et al. 2023). The purpose of choosing *Fusarium*, not *Neocosmospora* or other generic names is to maintain the stability of the name *Fusarium* in plant pathology and minimize confusion. We hope more independent studies in the future will resolve the phylogenetic disputes on *Fusariums. l.*

Morphology is a fundamental component of the generic and species concepts of fungi and must not be overlooked. Key morphological features for generic circumscription include characteristics of sexual morphs such as perithecial morphology, the presence and nature of a basal stroma, ascus characters, and ascospore shape, septation, color as well as surface ornamentation (Rossman et al. 1999), but sexual stage rarely develop. Therefore, diagnostic characters are the dimensions and characteristics of aerial conidiophores and conidiogenous cells (mono- vs. poly-phialides), presence/absence and characteristics of sporodochia, the types of conidia produced, e.g., aerial microconidia, and aerial and sporodochial macroconidia. Finally, the presence or absence of chlamydospores may be important (Leslie and Summerell 2006). However, the morphology of fungal structures will vary dramatically depending on the selection of media and growth conditions, which may compromise the identification process, and some *Fusarium* strains are similar in colony morphology and biology, which also makes it difficult to directly differentiate strains (Crous et al. 2021).

Current *Fusarium* taxonomy is dominated by molecular phylogenetic studies. Many protein-coding genes have been explored for identification and taxonomic purposes in *Fusarium*. The 28S large subunit (LSU) nrDNA, internal transcribed spacer region and intervening 5.8S nrRNA gene (ITS), large subunit of the ATP citrate lyase (acl1), RNA polymerase II largest subunit (*rpb1*), RNA polymerase II second largest subunit (*rpb2*), α-actin (*act*), β-tubulin (*tub2*), calmodulin (cmdA), histone H3 (*his3*), and translation elongation factor 1-alpha (tef1) loci are currently used (Lombard et al. 2015; Sandoval-Denis et al. 2018; Crous et al. 2021). However, *TEF-1α* and *RPB2* sequences appear to be the most useful in taxonomic studies of fungi of the *Fusarium* genus. Both offer high discriminatory power and are well represented in public databases (O’Donnell 2000). *TEF-1α* is commonly the first-choice identification marker as it has very good resolution power for most species, while *RPB2* allows for enhanced discrimination between closely related species (Crous et al. 2021). Additional genetic markers, often employed in association with the previously mentioned genes in multigene phylogenetic analyses, include *TUB2*, *HIS3*, *CAM*, and *RPB1*. These markers have variable resolution or applicability depending on the genus or species complex (Crous et al. 2021). One of the latest studies has used 19 loci to provide a much better phylogeny of *Fusarium* (Geiser et al. 2021). At present, Genealogical Concordance Phylogenetic Species Recognition (GCPSR) (Taylor et al. 2000) based multilocus data analyses have resolved *Fusarium* into >400 phylogenetically distinct species distributed among 23 monophyletic species complexes and several single-species lineages (O’Donnell et al. 2015; Summerell 2019; O’Donnell et al. 2020; Geiser et al. 2021).

Chinese fir (*Cunninghamialanceolata* (Lamb.) Hook.) is an evergreen coniferous tree species. Because of its fast growth, straight trunk, and high economic value, it is widely cultivated in the Yangtze River Basin and the southern Qinling Mountains in China. It is the main afforestation tree species in southern China. Average timber volume is estimated at 500–800 m^3^/ha, and in China, *C.lanceolata* contributes 40% of the total commercial timber production (Zheng et al. 2016). However, *C.lanceolata* is often damaged by many diseases and insect pests (Lan et al. 2015). Some common insect pests include *Semanotussinoauster*, *Callidiumvillosulum*, and *Lobesiacunninghamiacola* (Lan et al. 2015). *Bartaliniacunninghamiicola*, *Berkeleyomycesbasicola* (≡ *Thielaviopsisbasicola*), *Bipolarisoryzae*, *Bi.setariae*, *Ceratocystisacaciivora*, *Chalaropsis* sp., *Colletotrichumcangyuanense*, *C.fructicola*, *C.gloeosporioides*, *C.kahawae*, *C.karstii*, *C.siamense*, *Curvulariaspicifera*, *Cur.muehlenbeckiae*, *Ceratocystiscollisensis*, *Diaportheanhuiensis*, *Dia.citrichinensis*, *Dia.unshiuensis*, *Dia.hongkongensis*, *Discosiapini*, *Lophodermiumuncinatum*, *Nigrosporasphaerica*, *Rhizoctoniasolani*, Fusariumoxysporumf.pini, and *Fusarium* sp. have been reported as pathogens on *C.lanceolata* (Anonymous 1979; Kobayashi and Zhao 1987; Wang et al. 1995; Chen 2002; Lan et al. 2015; Liu et al. 2015; Xu and Liu 2017; Huang et al. 2018; Tian et al. 2019; Zhou and Hou 2019; Cui et al. 2020a, b; He et al. 2022; Li et al. 2022; Dai et al. 2023; Liao et al. 2023).

An investigation of fungal diseases on leaves of *C.lanceolata* covering its main cultivation regions of *C.lanceolata* in China was conducted from 2016 to 2020 (unpublished data) and samples of leaf blight were collected. The foliar symptoms ranged from leaf spots, anthracnose to leaf blight. The leaf blight disease mainly caused pale brown to brownish necrotic needles on *C.lanceolata*. Our preliminary study showed that a number of fungi were responsible for the foliar diseases of *C.lanceolata* in the field, including *Alternaria* spp., *Bipolaris* spp., *Colletotrichum* spp., *Curvularia* spp., *Fusarium* spp., and *Pestalotiopsis* spp. The main aim of the present study is to determine the *Fusarium* spp. associated with *C.lanceolata*.

## ﻿Materials and methods

### ﻿Isolation of the potential fungal pathogen

A total of 20 isolates of *Fusarium* spp. were isolated from leaf blight disease samples of *C.lanceolata*, which were collected in four provinces (Fujian, Guangxi, Guizhou, and Hunan) in China (Suppl. material 1: table S1). Small sections (2 × 3 mm) were cut from the margins of infected tissues and surface sterilized in 75% alcohol for 30 s, then in 1% sodium hypochlorite (NaOCl) for 90 s, followed by three rinses with sterile water (Huang et al. 2016), then blotted dry with sterilized filter paper, placed on 2% potato dextrose agar (PDA) Petri plates with 100 mg/L ampicillin, and then cultured for 3 days at 25 °C in the dark. Fungal isolates were purified with the monosporic isolation method described by Li et al. (2007) using the spores produced with liquid cultures. Single-spore isolates were maintained on PDA plates. The obtained isolates were stored in the Forest Pathology Laboratory at Nanjing Forestry University. Holotype specimens of new species from this study were deposited at the China Forestry Culture Collection Center (**CFCC**), Chinese Academy of Forestry, Beijing, China.

### ﻿DNA extraction, PCR amplification and sequencing

Genomic DNA of 20 isolates was extracted using a modified CTAB method (Damm et al. 2008). The fungal plugs of each isolate were grown on the PDA plates for 5 days and then collected in a 2 mL tube. Then, 500 µL of chloroform and 500 µL of hexadecyltrimethyl ammonium bromide (CTAB) extraction buffer (0.2 M Tris, 1.4 M NaCl, 20 mM EDTA, 0.2 g/L CTAB) were added into the tubes, which were placed in a shaker at 25 °C at 200 rpm for 2-h. The mixture was centrifuged at 15,800 × *g* for 5 min. Then, 300 µL of the supernatant was transferred into a new tube, and 600 µL of 100% ethanol was added. The suspension was centrifuged at 15,800 × *g* for 5 min. At that point, 600 µL of 70% ethanol was added into the precipitate. The suspension was centrifuged at 15,800 × *g* for 5 min, and the supernatant was discarded. The DNA pellet was dried and re-suspended in 30 µL ddH_2_O.

The polymerase chain reaction (PCR) amplification was carried out on the extracted DNA. *TEF-1α*, *RPB2*, and *RPB1* were amplified with the primer sets of EF1/EF2 (O’Donnell et al. 1998), 5f2/7cr (Liu et al. 1999), and Fa/G2R (O’Donnell et al. 2010), respectively. The primer sequences were listed in Suppl. material 1: table S2.

PCR was performed in a 30 μl reaction volume containing 2 μL of genomic DNA (*ca.* 200 ng/μL), 15 μL of 2× Taq Plus Master Mix (Dye Plus) (Vazyme P212-01), 1 µL of 10 μM forward primer, 1 µL of 10 μM reverse primer, and 11 μL of ddH_2_O. The parameters for PCR protocol were 94 °C for 4 min, followed by 34 cycles of 30 s at 94 °C, annealing at a suitable temperature for the 30 s for different loci: 55 °C for *TEF-1α*, *RPB2*, and *RPB1*, 72 °C for 60 s, and a final elongation step at 72 °C for 10 min. All DNA sequencing was performed at Shanghai Sangon Biotechnology Company (Nanjing, China). The sequences derived in this study were deposited in GenBank. GenBank accession numbers of all isolates used for phylogenetic analyses were listed in Table 1.

**Table 1.** Cultures, specimens and DNA accession numbers included in this study.

Species name	Culture/specimen^1^	Host	Country/area	GenBank/ENA accession number^2^		
				TEF–1α	RPB2	RPB1
***Fusariumfujikuroi* species complex**						
*F.acutatum*	CBS 402.97^T^ (Ex-type)	Unknown	India	KR071754	KT154005	MT010947
*F.agapanthi*	NRRL 54463^HT^ (Ex-holotype)	African lily	Australia and Italy	KU900630	KU900625	KU900620
	NRRL 54464^HT^	African lily	Australia and Italy	–	KU900627	KU900622
*F.ananatum*	CBS 118516^T^	Unknown	Unknown	–	KU604269	MT010937
*F.awaxy*	LGMF 1930^HT^	stalk, *Zeamays*	Brazil	MG839004	MK766941	–
*F.bactridioides*	CBS 100057^T^	*Pinusleiophylla*	Arizona, USA	KC514053	–	MT010939
*F.begoniae*	CBS 452.97^T^	Begonia elatior hybrid	Germany	KC514054	MT010964	–
*F.brevicatenulatum*	CBS 404.97^T^	*Strigaasiatica*	Madagascar	MT011005	MT010979	MT010948
	NRRL 25447^T^	Unknown	Unknown	MN193859	MN193887	–
*F.concentricum*	MUCL 55980	*Musa* sp.	China	LT574935	LT575016	–
	MUCL 55983	*Musa* sp.	China	LT574938	LT575019	–
	CBS 450.97^T^	*Musasapientum* fruit	Costa Rica	MT010992	MT010981	MT010942
	SJ1-10 *	Chinese fir	China	ON734385	ON734365	OR683264
	SJ1-10-1 *	Chinese fir	China	ON734386	ON734366	OR683265
	SJ1-10-2 *	Chinese fir	China	ON734387	ON734367	OR683266
	SJ1-10-3 *	Chinese fir	China	ON734388	ON734368	OR683267
*F.circinatum*	NRRL 25331^T^ = CBS 405.97	Monterrey pine tree	USA	AF160295	JX171623	–
*F.fujikuroi*	HJYB-4	*Zanthoxylumarmatum*	China	MT902140	MT902141	–
	MUCL 55986	*Musa* sp.	China	LT574941	LT575022	–
	CBS 221.76^T^	*Oryzasativa* culm	Taiwan	KR071741	KU604255	–
	HN43-17-1 *	Chinese fir	China	ON734397	ON734377	OR683276
	HN43-17-1-1 *	Chinese fir	China	ON734398	ON734378	OR683277
	HN43-17-1-2 *	Chinese fir	China	ON734399	ON734379	OR683278
	HN43-17-1-3 *	Chinese fir	China	ON734400	ON734380	OR683279
*F.lactis*	NRRL 25200^NT^ = CBS 411.97 (Ex-neotype)	*Ficuscarica*	USA	AF160272	–	MT010954
*F.mangiferae*	NRRL 25226^T^ = BBA 69662	*Mangiferaindica*	India	AF160281	JX171622	–
*F.nygamai*	NRRL 13448^T^ = CBS 749.97	Necrotic sorghum root	Australia	AF160273	EF470114	MT010955
*F.pseudocircinatum*	NRRL 22946^T^ = CBS 126.73	*Solanum* sp.	Ghana	AF160271	–	MT010952
*F.pseudonygamai*	NRRL 13592^T^ = CBS 417.97	*Pennisetumtyphoides*	Nigeria	AF160263	–	MT010951
*F.ramigenum*	NRRL 25208^T^ = CBS 418.97	*Ficuscarica*	USA	AF160267	KF466412	MT010959
*F.sacchari*	NRRL 13999 = CBS 223.76	*Saccharumofficinarum*	India	AF160278	JX171580	–
*F.subglutinans*	NRRL 22016^T^ = CBS 747.97	Corn	USA	AF160289	JX171599	–
*F.thapsinum*	NRRL 22045 = CBS 733.97	*Sorghumbicolor*	South Africa	AF160270	JX171600	–
*F.udum*	NRRL 22949 = CBS 178.32	unknown	Germany	AF160275	–	–
*F.xyrophilum*	NRRL 62721	*Xyris* spp.	Guyana	–	MN193905	MW402721
	NRRL 62710	*Xyris* spp.	Guyana	–	MN193903	MW402720
*F.zealandicum* (Outgroup)	CBS 111.93^T^	*Hoheriapopulnea* bark	New Zealand	HQ728148	HM626684	–
***F.lateritium* species complex**						
*F.cassiae*	MFLUCC 18-0573^HT^	*Cassiafistula*	Thailand	MT212205	MT212197	–
*F.citri-sinensis*	YZU 191316^T^	*Citrussinensis* fruit	China	MW855826	MW855854	–
	YZU 181391	*Citrussinensis* fruit	China	MW855825	OM913582	–
*F.fujianense*	LC14 *	Chinese fir	China	ON734389	ON734369	OR683268
	LC14-1 *	Chinese fir	China	ON734390	ON734370	OR683269
*F.fujianense*	LC14-2 *	Chinese fir	China	ON734391	ON734371	OR683270
	LC14-3 *	Chinese fir	China	ON734392	ON734372	OR683271
*F.lateritium*	NRRL 52786	unknown	Germany	JF740854	JF741180	JF741009
*F.lateritium*	NRRL 25122^LT^ (Ex-lectotype)	unknown	Germany	JF740747	JF741075	JF740959
*F.magnoliae-champaca*	MFLUCC 18-0580^HT^	*Magnoliachampaca*	Thailand	–	MT212198	–
*F.massalimae*	URM 8239^T^	*Handroanthuschrysotrichus*	Brazil	MN939763	MN939767	–
	FCCUFG 05^HT^	*Handroanthuschrysotrichus*	Brazil	MN939764	MN939768	–
*F.sarcochroum*	CPC 28118	*Citruslimon*	Castellò, Spain	LT746213	LT746326	LT746298
	CPC 28075^NT^	*Citrusreticulata*	Alginet, Spain	LT746211	LT746324	LT746296
*F.stilboides*	CBS 746.79^T^	*Citrus* sp.	New Zealand	MW928843	MW928832	–
*F.sublunatum* (Outgroup)	CBS 189.34^T^	*Musasapientum* and *Theobromacacao*	USA	–	KM232380	–
***F.sambucinum* species complex**						
*F.acaciae-mearnsii*	NRRL 26754^T^	*Acaciamearnsii*	South Africa	AF212448	KM361658	KM361640
*F.aethiopicum*	NRRL 46718	wheat seed	Ethiopia	FJ240296	KM361670	KM361652
	NRRL 46726	wheat seed	Ethiopia	MW233126	MW233470	MW233298
	NRRL 6227	*Triticumaestivum*	New South Wales, Australia	HM744692	JX171560	JX171446
	FRC R09335	*Triticumaestivum*	New South Wales, Australia	GQ915501	GQ915485	–
*F.concentricum* (Outgroup)	CBS 450.97^T^	*Musasapientum* fruit	Costa Rica	–	MT010981	MT010942
*F.cortaderiae*	NRRL 29297	*Cortaderia* sp.	New Zealand	MW233098	MW233442	MW233270
*F.culmorum*	NRRL 25475^T^	Barley	Denmark	MW233082	MW233425	MW233253
*F.guizhouense*	GZ7-20-1 *	Chinese fir	China	ON734381	ON734361	OR683260
	GZ7-20-1-1 *	Chinese fir	China	ON734382	ON734362	OR683261
	GZ7-20-1-2 *	Chinese fir	China	ON734383	ON734363	OR683262
	GZ7-20-1-3 *	Chinese fir	China	ON734384	ON734364	OR683263
*F.graminearum*	NRRL 31084	unknown	unknown	MW233103	JX171644	JX171531
*F.langsethiae*	NRRL 53439	oat kernel	Norway	HM744691	HQ154479	–
*F.longipes*	NRRL 20695	soil	USA	GQ915509	GQ915493	–
*F.louisianense*	NRRL 54197	*Triticumaestivum*	USA	KM889633	MW233478	MW233306
*F.mesoamericanum*	NRRL 25797	*Musa* sp.	Honduras	AF212441	MW233426	MW233254
*F.poae*	LC6917	*Oryzasativa*	China	MW620088	MW474613	MW024655
	LC13783	*Hordeumvulgare*	China	MW620087	MW474612	MW024654
	NRRL 26941^T^	Barley	USA	–	KU171706	KU171686
*F.pseudograminearum*	NRRL 28062^HT^	Unknown	Unknown	MW233090	JX171637	JX171524
*F.sambucinum*	MAFF 150447	Squash	Japan	LC637559	LC637561	–
	CBS 146.95^HT^	*Solanumtuberosum*	United Kingdom	KM231941	KM232381	–
*F.sibiricum*	NRRL 53432	Oat	Russia	HM744686	HQ154474	–
	NRRL 53430	Oat	Russia	HM744684	MW233474	MW233302
*F.sporotrichioides*	CBS 131779	*Avenasativa*	Canada	JX119003	JX162545	–
*F.transvaalense*	LLC3337	Soil	Australia	OP487291	OP486855	OP486422
	NRRL 31008	Soil	Australia	MW233102	MW233446	MW233274
*F.venenatum*	CBS 458.93^T^	*Winter wheat*	Australia	KM231942	KM232382	–
	NRRL 25413	Unknown	United Kingdom	MW233080	MW233423	MW233251
***F.solani* species complex**						
*F.ambrosium*	NRRL 22346	*Euwallaceafornicatus*	India	FJ240350	EU329503	KC691587
	NRRL 20438	*Euwallaceafornicatus*	India	AF178332	JX171584	JX171470
*F.bataticola*	CBS 144397	*Ipomoeabatatas*	USA	AF178343	EU329509	MW218099
	CBS 144398^T^	*Ipomoeabatatas*	USA	AF178344	FJ240381	MW218100
*F.borneense*	CBS 145462	Bark or recently dead tree	Indonesia	AF178352	EU329515	MW834213
*F.breviconum*	CBS 203.31	Twig	Philippines	LR583599	LR583820	MW218103
*F.cicatricum* (Outgroup)	CBS 125552	Dead twig	Slovenia	HM626644	HQ728153	–
*F.cryptoseptatum*	CBS 145463^T^	Bark	French Guiana	AF178351	EU329510	MW834215
*F.cucurbiticola*	CBS 410.62	*Cucurbitaviciifolia*	Netherlands	DQ247640	LR583824	MW834216
	CBS 616.66^T^	*Cucurbitaviciifolia*	Netherlands	DQ247592	LR583825	MW834217
*F.euwallaceae*	CBS 135854^T^	*Euwallacea* sp. on *Perseaamericana*	Israel	JQ038007	JQ038028	JQ038021
	NRRL 62626	*Euwallacea* sp. on *Perseaamericana*	USA	KC691532	KU171702	KU171682
*F.haematococcum*	CBS 119600^ET^	Dying tree	Sri Lanka	DQ247510	LT960561	–
*F.helgardnirenbergiae*	CBS 145469^T^	Bark	French Guiana	AF178339	EU329505	–
*F.hunanense*	HN33-8-2 *	Chinese fir	China	ON734393	ON734373	OR683272
	HN33-8-2-1 *	Chinese fir	China	ON734394	ON734374	OR683273
	HN33-8-2-2 *	Chinese fir	China	ON734395	ON734375	OR683274
	HN33-8-2-3 *	Chinese fir	China	ON734396	ON734376	OR683275
*F.illudens*	NRRL 22090	*Beilschmiediatawa*	New Zealand	AF178326	JX171601	JX171488
*F.kuroshium*	CBS 142642^T^	*Euwallacea* sp. on *Platanusracemosa*	USA	KX262216	LR583837	MW834227
*F.kurunegalense*	CBS 119599^T^	Recently cut tree	Sri Lanka	DQ247511	LR583838	MW834228
*F.lichenicola*	CBS 279.34^T^	Human	Somalia	LR583615	LR583840	–
*F.mahasenii*	CBS 119594^T^	Dead branch on live tree	Sri Lanka	DQ247513	LT960563	MW834231
*F.neocosmosporiellum*	CBS 446.93^T^	Soil	Japan	LR583670	LR583898	MW834257
*F.oligoseptatum*	CBS 143241^T^	*Euwallaceavalidus* on *Ailanthusaltissima*	USA	KC691538	LR583854	–
	NRRL 62578	*Euwallaceavalidus* on *Ailanthusaltissima*	USA	KC691537	KC691626	KC691595
*F.phaseoli*	NRRL 31041^T^	*Glycinemax*	USA	AY220193	JX171643	JX171530
*F.piperis*	CBS 145470^T^	*Pipernigrum*	Brazil	AF178360	EU329513	MW834241
*F.plagianthi*	NRRL 22632	*Hoheriaglabrata*	New Zealand	AF178354	JX171614	JX171501
*F.protoensiforme*	CBS 145471^T^	Dicot tree	Venezuela	AF178334	EU329498	MW834244
*F.pseudensiforme*	CBS 130.78	*Cocosnucifera*	Indonesia	DQ247635	LR583868	MW834245
	CBS 125729^T^	Dead tree	Sri Lanka	KC691555	KC691645	KC691615
*F.rectiphorum*	CBS 125727^T^	Dead tree	Sri Lanka	DQ247509	LR583871	MW834249
*F.samuelsii*	CBS 114067^T^	Bark	Guyana	LR583644	LR583874	MW834252
*F.staphyleae* (Outgroup)	NRRL 22316	*Staphyleatrifolia*	USA	AF178361	EU329502	JX171496
*Fusarium* sp.	YZU 171871	*Citrussinensis*	China	MK370098	MK370099	–
	YZU 171870	*Citrussinensis*	China	MH423886	MH423885	–
*F.venezuelense*	CBS 145473^T^	Bark	Venezuela	AF178341	EU329507	–
*F.xiangyunensis*	ZF-2018	Soil	China	MH992629	–	–
*F.yamamotoi*	CBS 144395	*Xanthoxylumpiperitum* branch	Japan	AF178328	EU329496	MW218112
	CBS 144396^ET^	*Xanthoxylumpiperitum* trunk	Japan	AF178336	FJ240380	MW218113

^1^ BBA: Biologische Bundesanstalt für Land- und Forstwirtschaft, Institut für Mikrobiologie, Berlin, Germany; CBS: Westerdijk Fungal Biodiverity Institute (WI), Utrecht, The Netherlands; CPC: Collection of P.W. Crous, held at WI; HMAS: Herbarium Mycologicum Academiae Sinicae, Chinese Academy of Sciences, Beijing, China; NRRL: Agricultural Research Service Culture Collection, National Center for Agricultural Utilization Research, USDA, Peoria, IL, USA; URM: the University Recife Mycology culture collection at the Universidade Federal de Pernambuco, Recife, Brazil; FCCUFG: Fungal Culture Collection of the Universidade Federal de Goiás; FRC: Fusarium Research Center, University Park, PA, USA; MUCL: Mycotheque de lUniversite Catholique de Louvain, Louvain-la-Neuve, Belgium; ^ET^: Ex-epitype, ^LT^: Ex-lectotype, ^NT^: Ex-neotype, ^HT^: Ex-holotype, ^T^: Ex-type, *: Sequences generated in this study. ^2^TEF-1α: translation elongation factor 1-alpha; RPB2: RNA polymerase second largest subunit; RPB1: RNA polymerase largest subunit.

### ﻿Phylogenetic analyses

The sequences generated in this study were compared against nucleotide sequences in GenBank using BLAST to determine closely related taxa. Alignments of different loci, including the sequences obtained from this study and sequences downloaded from the GenBank, were initially performed with the MAFFT v.7 online server (https://mafft.cbrc.jp/alignment/server/) (Katoh and Standley 2013) and then manually adjusted in MEGA v. 10 (Kumar et al. 2018). The post-alignment sequences of multiple loci were concatenated in PhyloSuite software (Zhang et al. 2020). Maximum Likelihood (ML) and Bayesian Inference (BI) analyses were conducted with PhyloSuite software using IQ-TREE ver. 1.6.8 (Nguyen et al. 2015) and MrBayes v. 3.2.6 (Ronquist et al. 2012), respectively. ModelFinder was used to carry out statistical selection of best-fit models of nucleotide substitution using the corrected Akaike information criterion (AIC) (Kalyaanamoorthy et al. 2017) (Suppl. material 1: table S3). For ML analyses the default parameters were used and bootstrap support (BS) was carried out using the rapid bootstrapping algorithm with the automatic halt option. Bayesian analyses included two parallel runs of 2,000,000 generations, with the stop rule option and a sampling frequency set to each 1,000 generations. The 50% majority rule consensus trees and posterior probability (PP) values were calculated after discarding the first 25% of the samples as burn-in. Phylogenetic trees were visualized in FigTree v. 1.4.2 (http://tree.bio.ed.ac.uk/software/figtree/) (Rambaut 2014).

Phylogenetically related but ambiguous species were analyzed using the genealogical concordance phylogenetic species recognition (GCPSR) model by performing a pairwise homoplasy index (PHI) test as described by Quaedvlieg et al. (2014). The PHI test was performed in SplitsTree4 (Huson 1998; Huson and Bryant 2006) in order to determine the recombination level within phylogenetically closely related species using a concatenated multi-locus dataset (*TEF-1α*, *RPB2* and *RPB1*). If the pairwise homoplasy index results were below a 0.05 threshold (Ф_w_ < 0.05), it indicates significant recombination present in the dataset. The relationship among the closely related species was visualized by constructing splits graphs.

### ﻿Morphological study

One representative isolate was randomly selected from each *Fusarium* species for morphological research according to the method of Leslie and Summerell (2006). The isolates were transferred from the actively growing edge of a 4-day old colony by cutting mycelial blocks (6 mm in diameter), plated on to fresh potato dextrose agar (PDA) (Crous et al. 2021), oatmeal agar (OMA) (Crous et al. 2021), corn meal agar (CMA) (Thompson et al. 2013), and synthetic nutrient-poor agar (SNA) (Crous et al. 2021) plates and incubated at 25 °C in the dark. Alternatively, the isolates were also plated on to carnation leaf agar (CLA) (Crous et al. 2021) to induce sporulation when this failed on other media. The growth rate was recorded by measuring the diameter of the colonies until day 5, and the mean growth rate was calculated per day. The colony characters including colony color, texture, and pigment production were also recorded. The morphology and size of ascomata and conidiomata were studied and recorded using a Zeiss stereo microscope (SteRo Discovery v20). The shape, color and size of conidiophores, conidia were observed using a ZEISS Axio Imager A2m microscope (ZEISS, Germany) with differential interference contrast (DIC) optics. At least 30 measurements per structure were performed using Carl Zeiss Axio Vision software to determine their sizes, unless no or fewer individual structures were produced.

### ﻿Pathogenicity tests

The fungal isolates HN43-17-1, SJ1-10, LC14, GZ7-20-1, and HN33-8-2 were randomly selected from the *Fusarium* species for Koch’s postulates test. A conidial suspension of 10^6^ conidia/ml of each isolate was used for inoculation.

For *in vitro* inoculation, healthy young leaves of *C.lanceolata* were collected from 1-year-old *C.lanceolata* plants on the campus of Nanjing Forestry University, Jiangsu, China. Detached leaves were surface-sterilized with 75% ethanol, washed three times with sterile water, and air-dried on sterile filter paper. A 10 μl aliquot of conidial suspension was transferred to a sterile plastic tube (6 mm diameter, 20 mm deep), in which a leaf was placed so that the base of the leaf was immersed in the conidial suspension. The control was treated with the same amount of double-distilled water. Leaves in the tubes were then put in plastic trays (40 × 25 cm), covered with a piece of plastic wrap to maintain relative humidity at 99%, and incubated at 25 °C in the dark for 5 days. Each treatment had eight replicates, and the experiment was conducted three times. Symptom development on the detached leaves was evaluated by determining the means of lesion lengths at 5 days post inoculation (dpi). The data were analyzed by analysis of variance (ANOVA) using SPSS v. 18 software. LSD’s range test was used to determine significant differences among or between different treatments (Chung et al. 2020). Origin v. 8.0 software was used to draw histograms (Li et al. 2020).

For *in vivo* inoculation, shoots from *C.lanceolata* tissue culture seedlings provided by Fujian Yangkou Forest Farm, Fujian, China were used. Fifty-four bottles of seedlings (cultured with 0.6% water agar medium, one seedling per bottle) were prepared. A 10 µl aliquot of conidial suspension was applied onto each of the leader shoots. The same volume of distilled water was used as a control. After inoculation, the seedlings were incubated at 28 °C with a 12-h/12-h light/dark photoperiod for 10 days. The experiment was conducted three times, and each treatment had three replicates. Pathogens were re-isolated from the resulting lesions and identified as afore-described.

## ﻿Results

### ﻿Phylogenetic analyses

**Figure 1.**
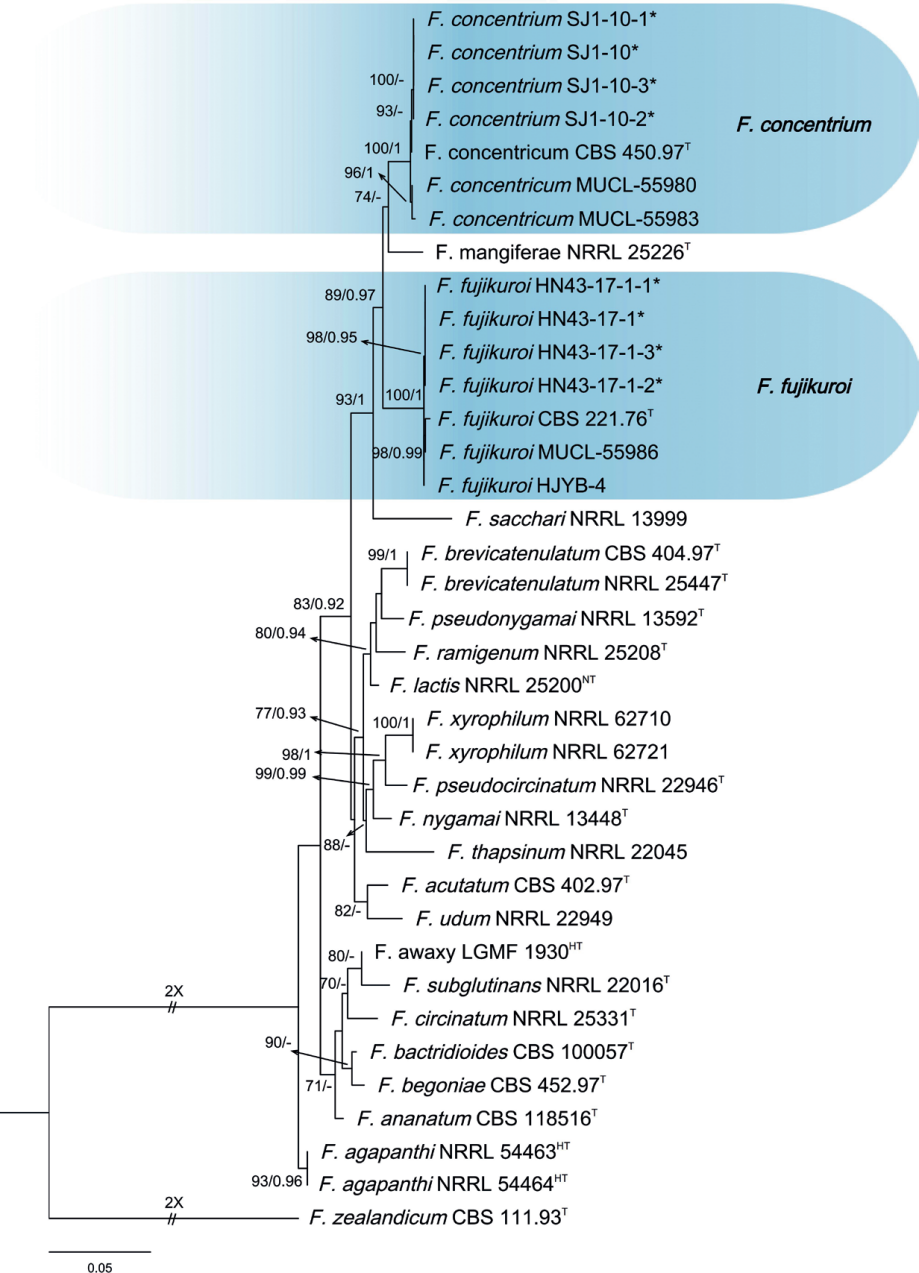
Phylogenetic relationships of 37 isolates of the *Fusariumfujikuroi* species complex with related taxa derived from concatenated sequences of the *TEF-1α*, *RPB2*, and *RPB1* genes/region using Bayesian inference (BI) and maximum likelihood (ML) methods. Bootstrap support values from ML ≥ 70% and BI posterior values ≥ 0.9 are shown at nodes (ML/BI). *Fusariumzealandicum* CBS 111.93^T^ was the outgroup. * indicates strains of this study. ^T^ indicates ex-types or ex-epitypes. ^LT^: Ex-lectotype, ^NT^: Ex-neotype, ^HT^: Ex-holotype.

A total of 20 *Fusarium* isolates were isolated from the diseased *C.lanceolata* samples showing the symptom of leaf blight and used for phylogenetic analyses. Three-locus phylogenetic analysis used 37 isolates of 22 related taxa from the *F.fujikuroi* species complex. *Fusariumzealandicum* CBS 111.93 (ex-type) was used as the out-group. A total of 2219 characters (*RPB1*: 1-901, *RPB2*: 902-1692, *TEF-1α*: 1693-2219) were included in the phylogenetic analyses. The Bayesian Inference (BI) and Maximum-likelihood (ML) phylogenetic analyses of the isolates of *F.fujikuroi* species complex produced topologically similar trees. The BI posterior probabilities (PP) were plotted on the ML tree (Fig. 1). In the combined analyses, four isolates (SJ1-10, SJ1-10-1, SJ1-10-2, and SJ1-10-3) were placed in the same clade with *F.concentricum* with high support (ML-BS/BI-PP = 100/1). Four isolates (HN43-17-1, HN43-17-1-1, HN43-17-1-2, and HN43-17-1-3) clustered in *F.fujikuroi* clade with high supports (ML-BS/BI-PP = 100/1).

**Figure 2.**
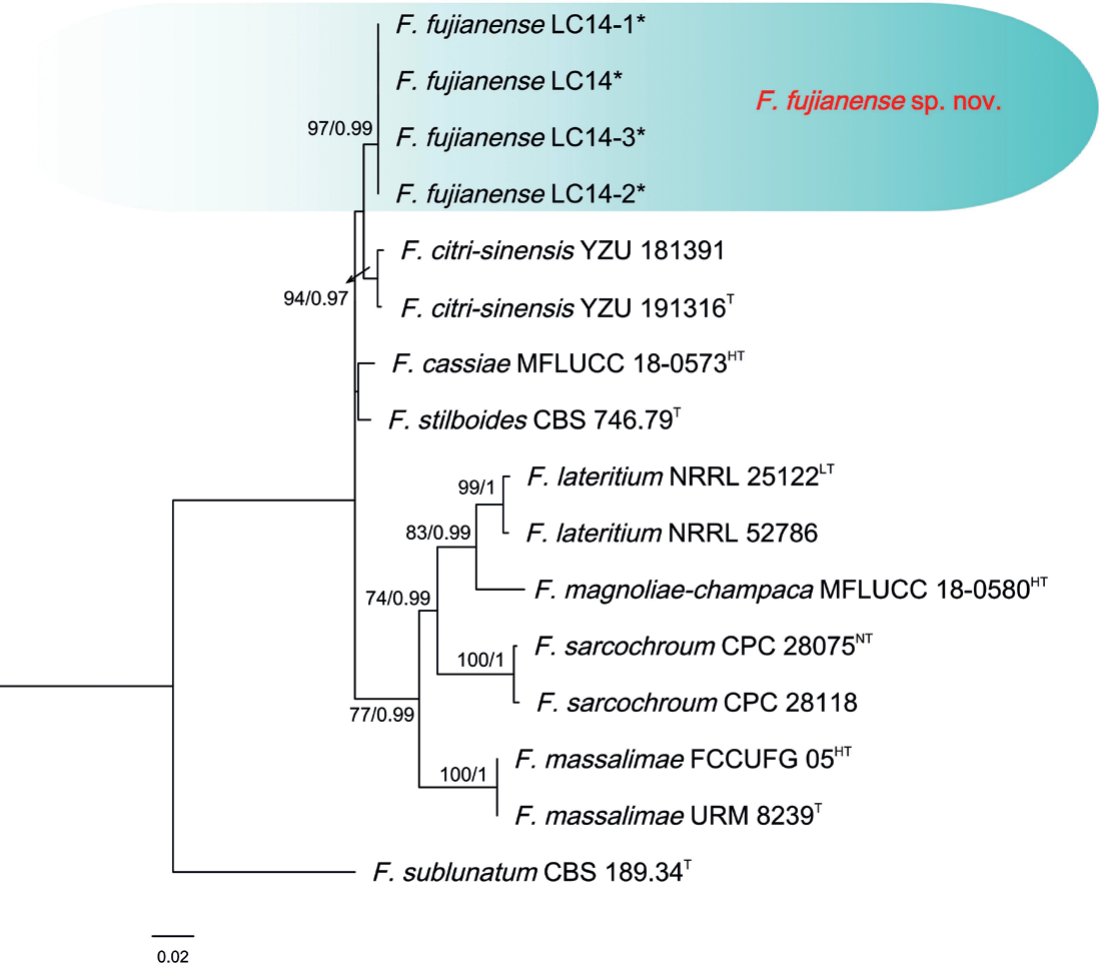
Phylogenetic relationships of 16 isolates of the *Fusariumlateritium* species complex with related taxa with concatenated sequences of the *TEF-1α*, *RPB2*, and *RPB1* loci using Bayesian inference (BI) and maximum likelihood (ML) methods. Bootstrap support values from ML ≥ 70% and BI posterior values ≥ 0.9 are shown at nodes (ML/BI). *Fusariumsublunatum* CBS 189.34^T^ was the outgroup. * indicates strains of this study. ^T^ indicates the ex-type strains. ^LT^: Ex-lectotype, ^NT^: Ex-neotype, ^HT^: Ex-holotype.

**Figure 3.**
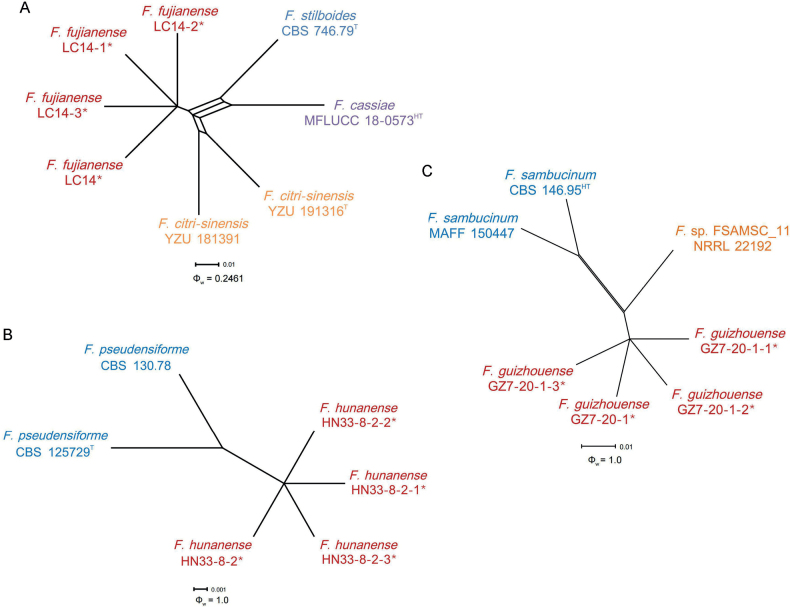
Splitgraphs showing the results of the pairwise homoplasy index (PHI) test of three newly described taxa and closely related species using both LogDet transformation and splits decomposition **A** the PHI of *Fusariumfujianense* sp. nov. with their phylogenetically related isolates or species **B** the PHI of *F.hunanense* sp. nov. with their phylogenetically related isolates or species **C** the PHI of *F.guizhouense* sp. nov. with their phylogenetically related isolates or species. PHI test value (Φ_w_) < 0.05 indicate significant recombination within a dataset. * indicates isolates of this study. ^T^ indicates ex-types. ^HT^ indicates ex-holotypes.

The three-locus phylogenetic analysis used 16 isolates of 8 related taxa from the *F.lateritium* species complex. *Fusariumsublunatum* CBS 189.34 (ex-type) was used as the out-group. A total of 2063 characters (*RPB1*: 1-615, *RPB2*: 616-1391, *TEF-1α*: 1392-2063) were included in the phylogenetic analyses. The Bayesian Inference (BI) and Maximum-likelihood (ML) phylogenetic analyses of the isolates of *F.lateritium* species complex produced topologically similar trees. The BI posterior probabilities (PP) were plotted on the ML tree (Fig. 2). Phylogenetic analyses showed that the four isolates (LC14, LC14-1, LC14-2, and LC14-3) clustered in a distinct clade with high supports (ML-BS/BI-PP = 97/0.99), which was distinct from all other known species and closely related to *F.citri-sinensis* (ex-type, YZU 191316), *F.cassiae* (ex-holotype, MFLUCC 18-0573), *F.stilboides* (ex-type, CBS 746.79) (Fig. 2). When applying the GCPSR concept to these isolates, the concatenated sequence dataset of three-loci (*TEF-1α*, *RPB2*, and *RPB1*) was subjected to the PHI test showed that no significant recombination was detected among these isolates/taxa (Φ_w_ = 0.2461) (Fig. 3A), which was a solid support for the proposition that these isolates belonged to four distinct taxa.

**Figure 4.**
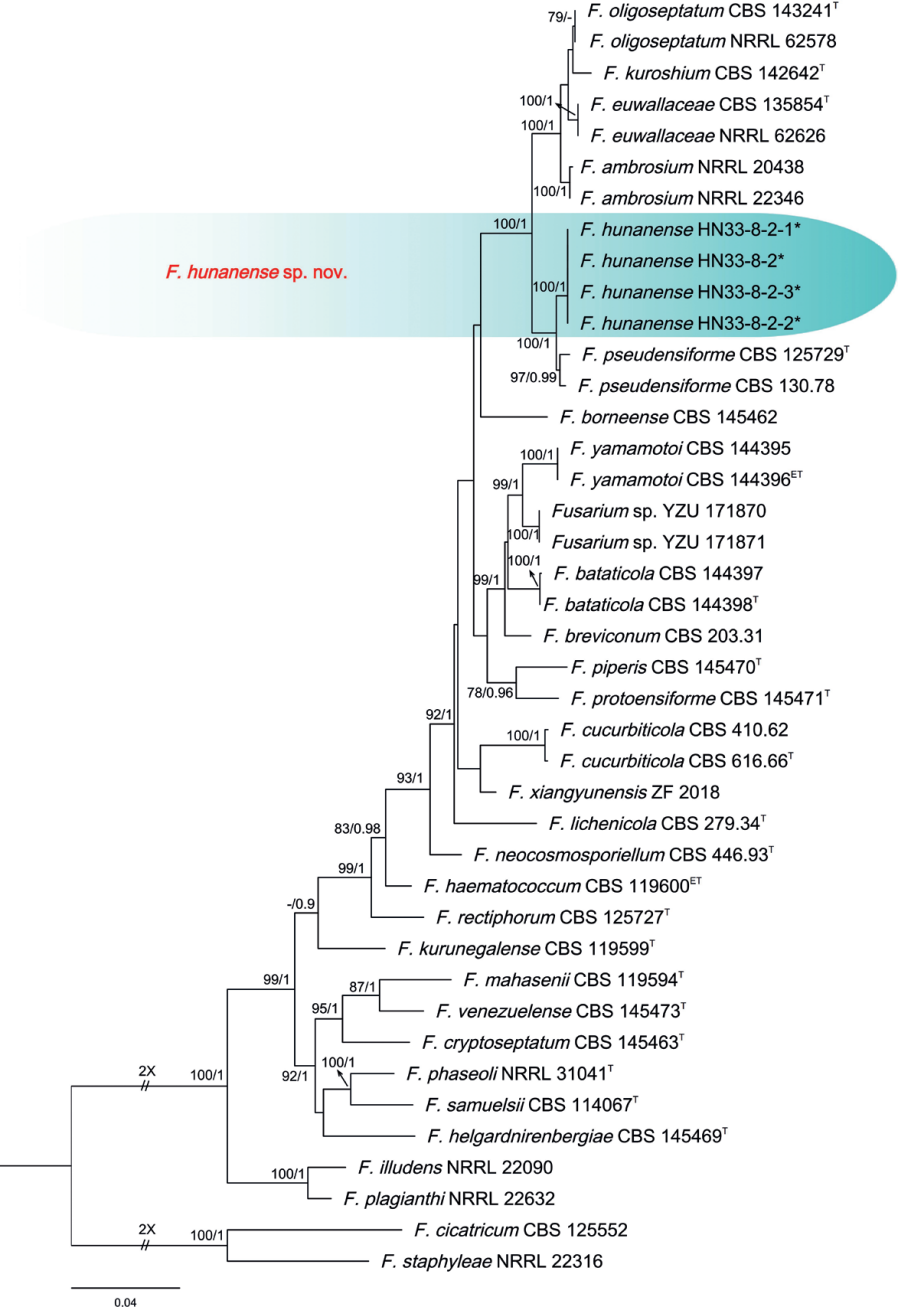
Phylogenetic relationships of 41 isolates of the *Fusariumsolani* species complex with related taxa with concatenated sequences of the *TEF-1α*, *RPB2*, and *RPB1* loci using Bayesian inference (BI) and maximum likelihood (ML) methods. Bootstrap support values from ML ≥ 70% and BI posterior values ≥ 0.9 are shown at nodes (ML/BI). *Fusariumstaphyleae* NRRL 22316 and *F.cicatricum* CBS 125552 were the outgroup. * indicates strains of this study. ^T^ indicates the ex-type strains. ^ET^ indicates ex-epitypes.

The three-locus phylogenetic analysis used 41 isolates of 29 related taxa from the *F.solani* species complex. *Fusariumstaphyleae* NRRL 22316 and *F.cicatricum* CBS 125552 were used as the out-group. A total of 2023 characters (*RPB1*: 1-640, *RPB2*: 641-1440, *TEF-1α*: 1441-2023) were included in the phylogenetic analyses. The Bayesian Inference (BI) and Maximum-likelihood (ML) phylogenetic analyses of the isolates of *F.solani* species complex produced topologically similar trees. The BI posterior probabilities (PP) were plotted on the ML tree (Fig. 4). Phylogenetic analyses showed that the four isolates (HN33-8-2, HN33-8-2-1, HN33-8-2-2, and HN33-8-2-3) clustered in a distinct clade with high supports (ML-BS/BI-PP = 100/1). These isolates were distinct from all other known species and closely related to *F.pseudensiforme* (ex-type, CBS 125729) (Fig. 4). When applying the GCPSR concept to this species, the concatenated sequence dataset of three-loci (*TEF-1α*, *RPB2*, and *RPB1*) was subjected to the PHI test showed that no significant recombination was detected among these isolates/taxa (Φw = 1.0) (Fig. 3B), which was a good support for the proposition that these isolates belonged to two distinct taxa.

**Figure 5.**
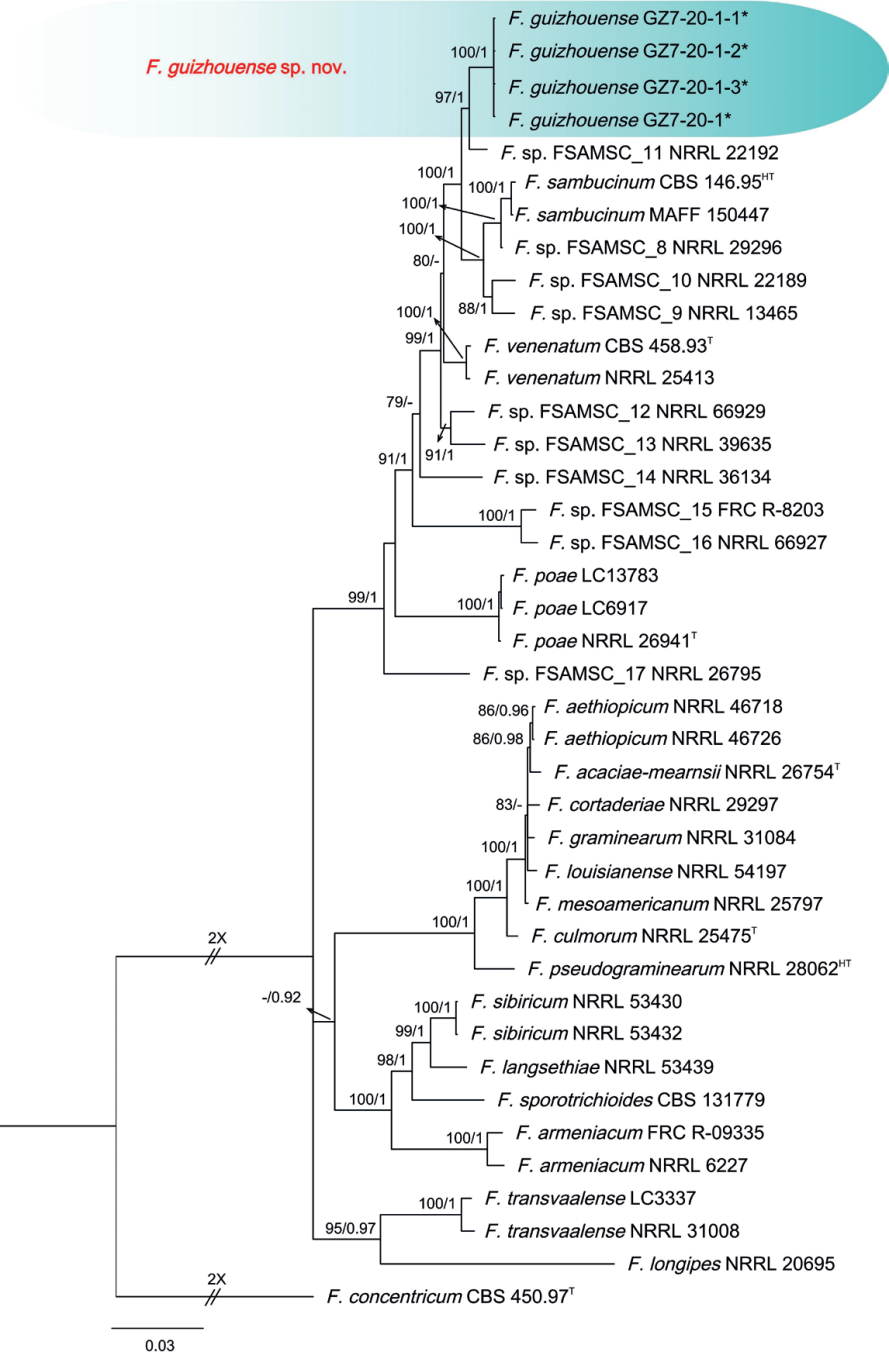
Phylogenetic relationships of 30 isolates of the *Fusariumsambucinum* species complex with related taxa with concatenated sequences of the *TEF-1α*, *RPB2*, and *RPB1* loci using Bayesian inference (BI) and maximum likelihood (ML) methods. Bootstrap support values from ML ≥ 70% and BI posterior values ≥ 0.9 are shown at nodes (ML/BI). *F.concentricum* CBS 450.97^T^ was the outgroup. * indicates strains of this study. ^T^ indicates the ex-type strains. ^HT^ indicates ex-holotypes.

The three-locus phylogenetic analysis used 30 isolates of 18 related taxa from the *F.sambucinum* species complex. *Fusariumconcentricum* CBS 450.97 (ex-type) was used as the out-group. A total of 2115 characters (*RPB1*: 1-641, *RPB2*: 642-1538, *TEF-1α*: 1539-2115) were included in the phylogenetic analyses. The Bayesian Inference (BI) and Maximum-likelihood (ML) phylogenetic analyses of the isolates of *F.sambucinum* species complex produced topologically similar trees. The BI posterior probabilities (PP) were plotted on the ML tree (Fig. 5). Phylogenetic analyses showed that the four isolates (GZ7-20-1, GZ7-20-1-1, GZ7-20-1-2, and GZ7-20-1-3) clustered in a distinct clade with high supports (ML-BS/BI-PP = 100/1), which was distinct from all other known species and identified as closely related to *F.venenatum* (ex-type, CBS 458.93), *F.poae* (ex-type, NRRL 26941), and *F.sambucinum* (ex-holotype, CBS 146.95) (Fig. 5). When applying the GCPSR concept to these isolates, the concatenated sequence dataset of three-loci (*TEF-1α*, *RPB2*, and *RPB1*) was subjected to the PHI test and showed that no significant recombination was detected among these isolates/taxa (Φ_w_ = 0.7313) (Fig. 3C). The split tree decomposition network of these multiple combinations was clearly detected within four separate groups.

### ﻿Taxonomy

The results of the molecular analyses and observations of morphological characteristics in culture indicated that the 20 isolates from *C.lanceolata* belonged to five *Fusarium* species, among which two were known taxa (*F.concentricum* and *F.fujikuroi*) and three were new to science (*F.fujianense*, *F.guizhouense*, and *F.hunanense*). This study selected the representative strains of each *Fusarium* species SJ1-10 (*F.concentricum*), LC14 (*F.fujianense*), HN43-17-1 (*F.fujikuroi*), GZ7-20-1 (*F.guizhouense*), and HN33-8-2 (*F.hunanense*) for detailed morphological characterization.

#### ﻿ Fusarium concentricum

Taxon classificationFungiHypocrealesNectriaceae

Nirenberg & O’Donnell, Mycologia 90 (3): 442 (1998)

470BFFF4-BD10-5E47-A3E1-EE4D09B8E0AF

MycoBank No: 444884

Suppl. material 1: fig. S1

##### Description.

Sexual state not observed. Asexual state: sporulation abundant from sporodochia, rarely from conidiophores formed directly on the substrate mycelium. Conidiophores in the aerial mycelium branched, bearing terminal or intercalary monophialides, often reduced to single phialides. Phialides subulate to subcylindrical, smooth, thin-walled, (2.3–)4.9–15.5(–18.3) × (1.1–)1.4–2.8(–3.5) μm, (mean ± SD = 10.2 ± 5.3 × 2.1 ± 0.7 μm, n = 9), without periclinal thickening. Microconidia in the aerial mycelium hyaline, ellipsoidal to falcate, smooth, thin-walled, 0–1-septate, (3.8–)5.9–9.1(–11.3) × (1.9–)2.5–3.4(–4.3) μm (mean ± SD = 7.5 ± 1.6 × 3.0 ± 0.5 μm, n = 60), forming small false heads on the tips of monophialides. Sporodochia pale orange colored, formed abundantly on carnation leaves. Conidiophores in sporodochia (27.7–)40.6–49.8(–51.7) μm, (mean ± SD = 45.2 ± 4.6 μm, n = 35), verticillately branched and densely packed, bearing apical whorls of 2–3 monophialides or rarely single lateral monophialides; sporodochial phialides subulate to subcylindrical, (9.5–)11.4–16.5(–20.4) × (2.2–)2.7–4.0(–4.7) μm, (mean ± SD = 13.9 ± 2.5 × 3.4 ± 0.6 μm, n = 45), smooth, thin-walled. Sporodochial macroconidia falcate, curved dorsiventrally with almost parallel sides tapering slightly towards both ends, with a blunt to papillate, curved apical cell and a foot cell, 3-septate, (23.2–)30.2–40.5(–43.7) × (3.4–)3.9–4.9(–5.5) μm, (mean ± SD = 35.3 ± 5.2 × 4.4 ± 0.5 μm, n = 60), 4-septate, (35.5–)38.0–48.8(–49.4) × (3.4–)3.4–4.3(–4.4) μm, (mean ± SD = 43.4 ± 5.4 × 3.9 ± 0.4 μm, n = 10), 5-septate, (49.5–)49.7–57.2(–59.1) × (3.5–)3.6–4.2(–4.2) μm, (mean ± SD = 53.4 ± 3.6 × 3.9 ± 0.3 μm, n = 10), hyaline, thin- and smooth-walled. Chlamydospores absent.

##### Culture characteristics.

Colonies on PDA growing in the dark with an average growth rate of 9.3 mm/d at 25 °C. Colony surface white to pale purple, flat or slightly raised at the center; colony margins irregular, filiform. Reverse light yellow. Odor absent. Colonies on SNA incubated at 25 °C in the dark were regular, round, aerial mycelium absent or scant, growing at 13.1 mm/d. Colonies on OMA incubated at 25 °C in the dark were regular, round, aerial mycelium abundant, loose to densely floccose, growing at 13.2 mm/d. Reverse light purple. Colonies on CMA incubated at 25 °C in the dark were regular, round, colony surface and reverse pale gray at the center, aerial mycelium absent or scarce, growing at 11.9 mm/d.

##### Materials examined.

China, Guangxi Zhuang Autonomous Region, Liuzhou City, Sanjiang Dong Autonomous County, Guyi Town, 25°25′48″N, 109°28′47″E, isolated from leaf spots of *Cunninghamialanceolata*, May 2017, Wen-Li Cui, isolates: SJ1-10, SJ1-10-1, SJ1-10-2, SJ1-10-3.

##### Notes.

The isolate SJ1-10 in this study was in the same clade with *F.concentricum* CBS 450.97 (ex-type). Morphologically, 0-septate microconidia (3.8–11.3 × 1.9–4.3 μm) of the isolate SJ1-10 were similar with the 0-septate microconidia (7.0–12.2 ×2.3–3.9 μm) of the ex-type (CBS 450.97) of *F.concentricum* (Nirenberg and O’Donnell 1998). Five-septate macroconidia (49.5–59.1 × 3.5–4.2 μm) of the isolate SJ1-10 were similar with the 5-septate macroconidia (49.0–64.8 × 3.6–4.0 μm) of the ex-type (CBS 450.97) of *F.concentricum* (Nirenberg and O’Donnell 1998).

#### ﻿ Fusarium fujikuroi

Taxon classificationFungiHypocrealesNectriaceae

Nirenberg, Mitteilungen der Biologischen Bundesanstalt für Land- und Forstwirtschaft 169: 32 (1976)

74AF3213-62C3-5011-AD8C-9113EF683F65

MycoBank No: 314213

Suppl. material 1: fig. S2

##### Description.

Sexual state not observed. Asexual state: Sporulation abundant from sporodochia, rarely from conidiophores formed directly on the substrate mycelium. Conidiophores in the aerial mycelium branched, bearing terminal or intercalary phialides. Phialides subulate to subcylindrical, smooth, thin-walled, (11.5–)14.7–22.9(–30.0) μm × (1.8–)2.0–3.6(–4.0) μm, (mean ± SD = 18.8 ± 4.1 μm × 2.8 ± 0.8 μm, n = 37), without periclinal thickening; microconidia hyaline, short clavate to cylindrical, slender to relatively straight, smooth, thin-walled, 0-septate, (5.4–)6.7–11.3(–15.5) × (2.0–)2.5–3.5(–4.4) μm, (mean ± SD = 9.0 ± 2.3 × 3.0 ± 0.5 μm, n = 81), forming small false heads on the tips of phialides. Chlamydospores formed occasionally, mostly in pairs or chains, terminal or intercalary, globose to subglobose, smooth-walled, (6.0–)6.2–8.0(–8.3) × (4.4–)4.4–5.2(–5.6) μm, (mean ± SD = 7.1 ± 0.9 × 4.8 ± 0.4 μm, n = 6). Sporodochia and macroconidia not observed.

##### Culture characteristics.

Colonies on PDA growing in the dark with an average growth rate of 13.9 mm/d at 25 °C. Colony surface white to purple, flat or slightly raised at the center; colony round, regular, margins filiform, aerial mycelium abundant. Reverse purple with white periphery. Odor absent. Colonies on SNA incubated at 25 °C in the dark were regular, round, growing at 8.1 mm/d. Colony surface pure white, aerial mycelium absent or scant. Reverse pure white, without diffusible pigments. Colonies on OMA incubated at 25 °C in the dark were regular, round, aerial mycelium abundant, loose to densely floccose, growing at 12.5 mm/d. Colony white to dark purple and with white to dark violet pigmentation. Colonies on CMA incubated at 25 °C in the dark were regular, round, colony surface and reverse white, aerial mycelium absent or scant, growing at 11.3 mm/d.

##### Materials examined.

China, Hunan province, Yiyang City, Heshan District, Henglongqiao Town, 28°27′24″N, 112°29′7″E, isolated from leaf spots of *Cunninghamialanceolata*, May 2017, Wen-Li Cui, isolates: HN43-17-1, HN43-17-1-1, HN43-17-1-2, HN43-17-1-3.

##### Notes.

The isolate HN43-17-1 in this study was in the same clade with *F.fujikuroi* CBS 221.76 (ex-type). Morphologically, 0-septate microconidia, (5.4–15.5 × 2–4.4 μm) of the isolate HN43-17-1 were more variable than the 0-septate microconidia (12.2–12.9 × 3.4–3.7 μm) of the ex-type (CBS 221.76) of *F.fujikuroi* (Ibrahim et al. 2016).

#### ﻿ Fusarium fujianense

Taxon classificationFungiHypocrealesNectriaceae

Lin Huang, Jiao He & D.W. Li sp. nov.

5FDB4F42-B17D-50FB-8F58-07567102848D

Index Fungorum Number: IF900473

Fig. 6

##### Etymology.

Epithet is after Fujian province where the type specimen was collected.

##### Holotype.

China, Fujian Province, Nanping City, Shunchang County, Yangkou Forest Farm, 26°48′36″N, 117°52′48″E, isolated from leaf spots of *Cunninghamialanceolata*, May 2017, Wen-Li Cui, (holotype: CFCC 57576). Holotype specimen is a living specimen being maintained via lyophilization at the China Forestry Culture Collection Center (CFCC). Ex-type (LC14) is maintained at the Forest Pathology Laboratory, Nanjing Forestry University.

**Figure 6.**
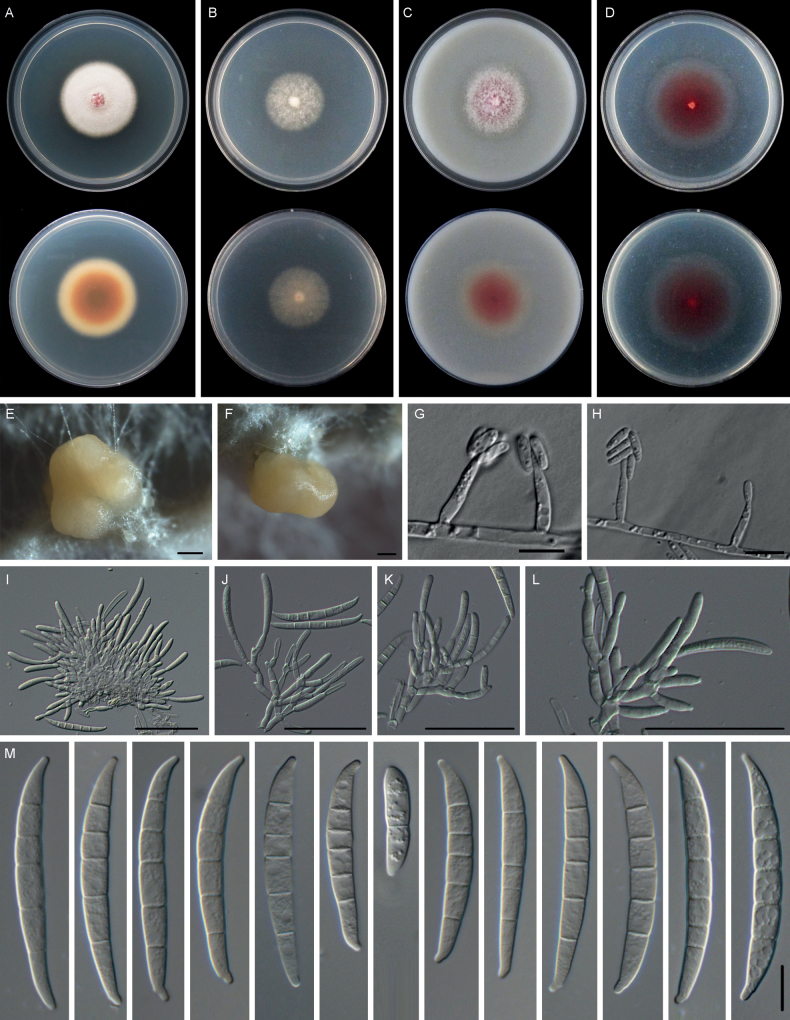
*Fusariumfujianense* (LC14) **A–D** colonies on PDA, SNA, OMA, and CMA, respectively, after 5 days at 24 °C in the dark **E, F** sporodochia formed on PDA**G, H** aerial conidiophores, phialides, and microconidia **I–L** sporodochial conidiophores, phialides, and macroconidia **M** mesoconidium (1-septate) and macroconidia (4–6-septate). Scale bars: 200 μm (**E, F**); 10 μm (**G–M**).

##### Host/distribution.

From *C.lanceolata* in Yangkou Forest Farm, Shunchang County, Nanping City, Fujian Province, China.

##### Description.

Sexual state not observed. Asexual state: Sporulation abundant from sporodochia, rarely from conidiophores formed directly on the substrate mycelium. Conidiophores in the aerial mycelium unbranched, bearing terminal or intercalary monophialides, often reduced to single phialides. Phialides subulate to subcylindrical, smooth, thin-walled, (9.2–)10.3–16.3(–18.0) μm × (2.5–)2.6–3.4(–3.6) μm, (mean ± SD = 13.3 ± 3.0 μm × 3.0 ± 0.4 μm, n = 11), without periclinal thickening; microconidia subcylindrical to clavate, hyaline, smooth- and thin-walled, 0-septate, (5.6–)6.0–8.2(–8.3) μm × (1.9–)2.1–2.5(–2.7) μm, (mean ± SD = 7.1 ± 1.1 μm × 2.3 ± 0.2 μm, n=11), forming small false heads on the tips of monophialides. Sporodochia pale orange colored, formed abundantly on PDA after 40 days. Conidiophores in sporodochia (9.7–)18.8–31.5(–37.9) μm, (mean ± SD = 25.1 ± 6.4 μm, n = 37), irregularly branched and densely packed, bearing apical whorls of monophialides or 2–3 ployphialides; sporodochial phialides subulate to subcylindrical, (5.6–)10.0–16.1(–18.8) × (1.4–)2.5–3.9(–4.8) μm, (mean ± SD = 12.7 ± 3.4 × 3.2 ± 0.7 μm, n = 39), smooth, thin-walled. Sporodochial mesoconidia falcate, curved dorsiventrally with almost parallel sides tapering slightly towards both ends, with a blunt to papillate, curved apical cell and a foot-like basal cell, 1-septate, (21.8–)22.0–23.6(–23.8) × (4.7–)4.9–5.3(–5.3) μm, (mean ± SD = 22.8 ± 0.8 × 5.1 ± 0.2 μm, n = 6), macroconidia 4–6-septate, (40.2–)45.9–59.1(–63.4) × (4.5–)4.8–5.8(–6.9) μm, (mean ± SD = 52.5 ± 6.6 × 5.3 ± 0.5 μm, n = 18), hyaline, smooth, thin-walled. Chlamydospores absent.

##### Culture characteristics.

Colonies on PDA growing in the dark with an average growth rate of 6.2 mm/d at 25 °C. Colony surface white to red, flat or slightly raised at the center; colony margins regular, round. Reverse red with white periphery. Odor absent. Colonies on SNA incubated at 25 °C in the dark were regular, round, growing at 5.4 mm/d. Colony surface pure white, aerial mycelium abundant. Reverse pure white, without diffusible pigments. Colonies on OMA incubated at 25 °C in the dark were regular, round, aerial mycelium abundant, loose to densely floccose, growing at 6.0 mm/d. Reverse red with white periphery. Colonies on CMA incubated at 25 °C in the dark were regular, round, colony surface and reverse red with white periphery, aerial mycelium absent or scant, growing at 7.1 mm/d.

##### Additional materials examined.

China, Fujian Province, Nanping City, Shunchang County, Yangkou Forest Farm, 26°48′36″N, 117°52′48″E, isolated from leaf spots of *Cunninghamialanceolata*, May 2017, Wen-Li Cui, isolates: LC14-1, LC14-2, LC14-3.

##### Notes.

The isolates of *F.fujianense* were phylogenetically closely related to *F.citri-sinensis* (ex-type, YZU 191316), *F.cassiae* (ex-holotype, MFLUCC 18-0573), and *F.stilboides* (ex-type, CBS 746.79) (Fig. 2). Between *F.fujianense* isolates and ex-type of *F.citri-sinensis* YZU 191316, there were 13/672 differences in *TEF-1α*, and 8/776 in *RPB2*. Between *F.fujianense* isolates and ex-holotype of *F.cassiae* MFLUCC 18-0573, there were 25/672 differences in *TEF-1α*, and 7/776 in *RPB2*. Between *F.fujianense* isolates and ex-type of *F.stilboides* CBS 746.79, there were 16/672 differences in *TEF-1α*, and 2/776 in *RPB2*. The *RPB1* sequences of *F.stilboides* CBS 746.79, *F.cassiae* MFLUCC 18-0573, and *F.citri-sinensis* YZU 191316 were missing. The PHI analysis showed that there was no significant recombination between *F.fujianense* isolates and its related species (Φw = 0.2461) (Fig. 3A). Morphologically, *F.fujianense* differed from *F.citri-sinensis* in colony characteristics on PDA. The former developed dense mycelia and abundant red pigmentation, while the latter was characterized by sparse and loose aerial mycelia and pale pink pigment (Zhao et al. 2022). *F.fujianense* can be differentiated from *F.cassiae* in having abundant red pigmentation produced in PDA vs. without diffusible pigments in *F.cassiae* (Perera et al. 2020). *F.fujianense* can be distinguished from *F.stilboides* by having different 0-septate conidia (5.6–8.3 × 1.9–2.7 μm vs. 7–14 × 2–2.5 µm) (Booth and Waterston 1964). Thus, *F.fujianense* is recognized as a novel species in *F.lateritium* species complex.

#### ﻿ Fusarium guizhouense

Taxon classificationFungiHypocrealesNectriaceae

Lin Huang, Jiao He & D.W. Li sp. nov.

6DE090D8-85D7-556C-B459-F6C6589E41CF

Index Fungorum Number: IF900474

Fig. 7

##### Etymology.

Epithet is after Guizhou Province where the type specimen was collected.

##### Holotype.

China, Guizhou Province, Qiandongnan Miao and Dong Autonomous Prefecture, Cengong County, Kelou Town, 27°22′58″N, 108°22′9″E, isolated from leaf spots of *Cunninghamialanceolata*, May 2017, Wen-Li Cui, (holotype: CFCC 57575). Holotype specimen is a living specimen maintained via lyophilization at the China Forestry Culture Collection Center (CFCC). Ex-type (GZ7-20-1) is maintained at the Forest Pathology Laboratory, Nanjing Forestry University.

**Figure 7.**
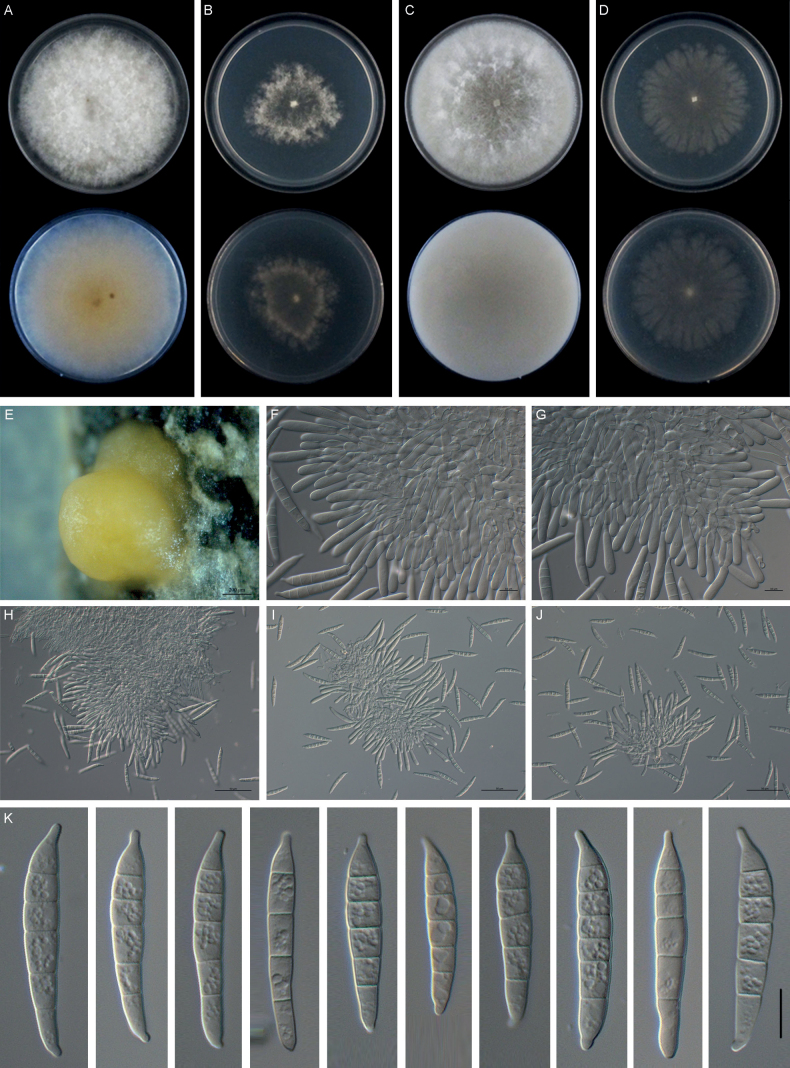
*Fusariumguizhouense* (GZ7-20-1) **A–D** colonies on PDA, SNA, OMA, and CMA, respectively, after 5 days at 24 °C in the dark **E** sporodochia formed on the surface of carnation leaves **F–J** sporodochial conidiophores, phialides, and macroconidia **K** macroconidia (4–6-septate). Scale bars: 200 μm (**E**); 10 μm (**F, G, K**); 50 μm (**H–J**).

##### Host/distribution.

From *C.lanceolata* in Kelou Town, Cengong County, Qiandongnan Miao and Dong Autonomous Prefecture, Guizhou Province, China.

##### Description.

Sexual state not observed. Asexual state: Sporulation abundant from sporodochia, rarely from conidiophores formed directly on the substrate mycelium. Conidiophores in the aerial mycelium absent. Sporodochia bright orange colored, formed abundantly on carnation leaves. Conidiophores in sporodochia (13.8–)18.8–25.8(–29.8) μm, (mean ± SD = 22.3 ± 3.5 μm, n = 39), irregularly branched and densely packed, bearing apical whorls of 1–4 phialides; sporodochial phialides subulate to subcylindrical, (8.2–)10.6–14.7(–16.9) × (2.7–)3.1–4.0(–4.8) μm, (mean ± SD = 12.6 ± 2.0 × 3.6 ± 0.5 μm, n = 40), smooth, thin-walled. Sporodochial macroconidia colorless, straight or slightly curved, wider at the middle or apical part, tapering towards the base, with a blunt and often curved apical cell and a foot-like to slightly notched basal cell, 4–5-septate. Four-septate conidia: (30.8–)33.3–40.9(–40.6) × (4.5–)5.3–6.4(–6.9) μm, (mean ± SD = 37.1 ± 3.8 × 5.9 ± 0.5 μm, n = 52), five-septate conidia: (33.4–)38.0–45.4(–51.3) × (5.0–)5.7–6.9(–7.5) μm, (mean ± SD = 41.7 ± 3.7 × 6.3 ± 0.6 μm, n = 60), smooth, thin-walled. Chlamydospores absent.

##### Culture characteristics.

Colonies on PDA growing in the dark with an average growth rate of 16.7 mm/d at 25 °C. Colony color white at first, becoming buff, felty to cottony. Aerial mycelium abundant, loose to densely floccose; margins irregular and fimbriate. Reverse pale buff with white periphery. Odor absent. Colonies on SNA incubated at 25 °C in the dark were irregular, growing at 9.7 mm/d. Colony surface pure white, aerial mycelium scant, forming irregular rings at the periphery of the colony; margins lobate or serrate. Reverse pure white, without diffusible pigments. Colonies on OMA incubated at 25 °C in the dark were irregular, aerial mycelium abundant, loose to densely floccose, growing at 13.1 mm/d. Colony in reverse was white with litter gray pigmentation. Colonies on CMA incubated at 25 °C in the dark were round, colony surface and reverse white, flat, radially striated, membranous to dusty, aerial mycelium scant or absent; colony margins irregular, lobate or serrate, growing at 9.6 mm/d.

##### Additional materials examined.

China, Guizhou province, Qiandongnan Miao and Dong Autonomous Prefecture, Cengong County, Kelou Town, 27°22′58″N, 108°22′9″E, isolated from leaf spots of *Cunninghamialanceolata*, May 2017, Wen-Li Cui, isolates: GZ7-20-1-1, GZ7-20-1-2, GZ7-20-1-3.

##### Notes.

The isolates of *F.guizhouense* were phylogenetically close to *F.sambucinum* (ex-holotype, CBS 146.95), *F.poae* (ex-type, NRRL 26941), and *F.venenatum* (ex-type, CBS 458.93) (Fig. 5). Between *F.guizhouense* isolates and ex-holotype of *F.sambucinum* CBS 146.95, there were 34/577 differences in *TEF-1α*, 8/897 in *RPB2*. The *RPB1* sequence of *F.sambucinum* CBS 146.95 was missing. Between *F.guizhouense* isolates and ex-type of *F.poae* NRRL 26941, there were 24/897 differences in *RPB2*, 26/641 in *RPB1*. The *TEF-1α* sequence of *F.poae* NRRL 26941 was missing. Between *F.guizhouense* isolates and ex-type of *F.venenatum* CBS 458.93, there were 20/577 differences in *TEF-1α*, 8/897 in *RPB2*. The *RPB1* sequence of *F.venenatum* CBS 458.93 was missing. The PHI analysis showed that there was no significant recombination between *F.guizhouense* isolates and its related species (Φ_w_ = 0.7313) (Fig. 3C). Morphologically, Sporodochial phialides of the *F.guizhouense* isolates (10.6–14.7 × 3.1–4.0 μm) were smaller than those of *F.sambucinum* NRRL 22203 (ex-lectotype) (14.0–18.0 × 3.8–4.5 µm) (Nirenberg 1995). *Fusarium* sp. FSAMSC_11 (NRRL 22192) is closely related to *F.guizhouense*, but it has no morphological data available (Laraba et al. 2021). Further study on this isolate (NRRL 22192) is necessary to determine its taxonomic placement. In conclusion, the phylogenetic and morphological evidence support this fungus being a new species within the *F.sambucinum* species complex.

#### ﻿ Fusarium hunanense

Taxon classificationFungiHypocrealesNectriaceae

Lin Huang, Jiao He & D.W. Li sp. nov.

D0D70022-1944-5506-A79E-318281D5606A

Index Fungorum Number: IF900475

Fig. 8

##### Etymology.

Epithet is named after Hunan Province where the type specimen was collected.

##### Holotype.

China, Hunan Province, Yiyang City, Heshan District, Henglongqiao Town, 28°27′24″N, 112°29′7″E, isolated from leaf spots of *Cunninghamialanceolata*, May 2017, Wen-Li Cui, (holotype: CFCC 57574). Holotype specimen is a living specimen maintained via lyophilization at the China Forestry Culture Collection Center (CFCC). Ex-type (HN33-8-2) is maintained at the Forest Pathology Laboratory, Nanjing Forestry University.

**Figure 8.**
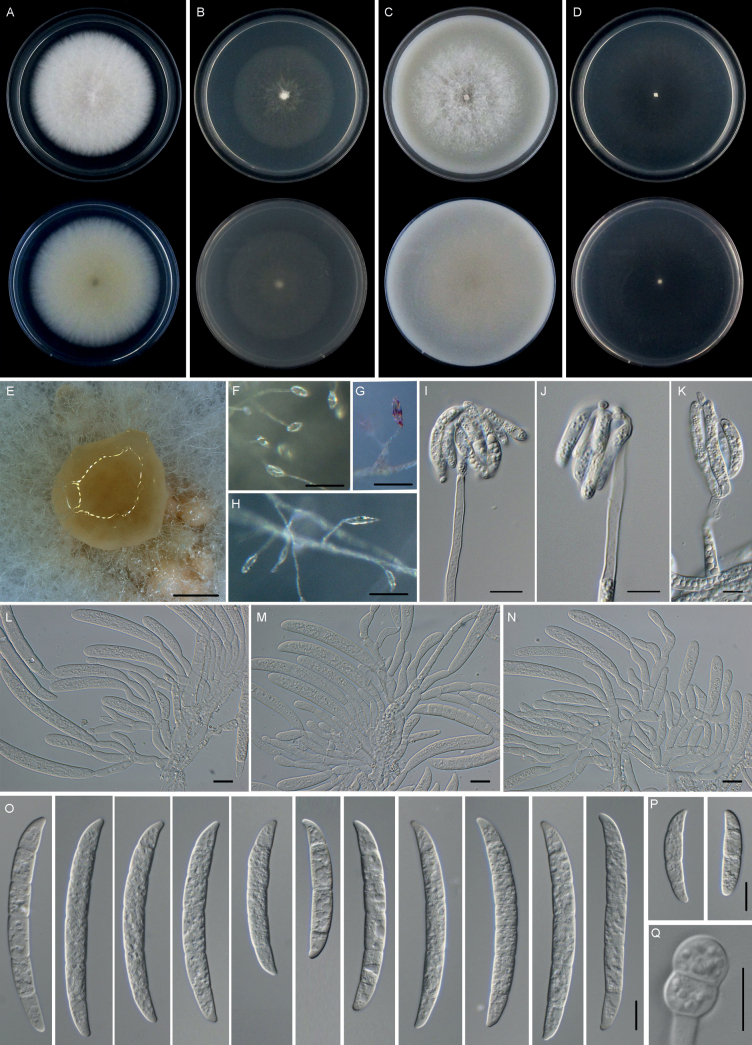
*Fusariumhunanense* (HN33-8-2) **A–D** colonies on PDA, SNA, OMA, and CMA, respectively, after 5 days at 24 °C in the dark **E** sporodochia formed on PDA**F–K** aerial conidiophores, phialides, and conidia **L–N** sporodochial conidiophores, phialides, and conidia **O, P** macroconidia (3–6-septate) **Q** chlamydospore. Scale bars: 1,000 μm (**E**); 50 μm (**F–H**); 10 μm (**I–Q**).

##### Host/distribution.

From *C.lanceolata* in Henglongqiao Town, Heshan District, Yiyang City, Hunan Province, China.

##### Description.

Sexual state not observed. Asexual state: sporulation abundant from erect conidiophores formed on the agar surface or aggregated in sporodochia. Conidiophores in the aerial mycelium, mostly unbranched, rarely basally dichotomously branched, forming monophialides on the apices; phialides slender, subulate to subcylindrical, monophialidic, smooth, thin-walled, (29.6–)31.6–54.6(–74.1) × (2.0–)2.2–2.8(–3.0) μm, (mean ± SD = 43.1± 11.5 × 2.5 ± 0.3 μm, n = 17), with slight periclinal thickening at the tip and a short flared apical collarette. Sporodochia cream colored, produced on the surface of carnation leaves and PDA medium. Conidiophores in sporodochia (26.0–)29.3–39.1(–46.8) μm, (mean ± SD = 34.1 ± 5.1 μm, n = 39), irregularly branched, short stipitate, occasionally in whorls bearing terminal 2–4 monophialides; sporodochial phialides subulate to subcylindrical, smooth, thin-walled, (11.4–)15.5–22.1(–28.6) × (3.3–)4.0–5.2(–6.0) μm, (mean ± SD = 18.8 ± 3.3 × 4.6 ± 0.6 μm, n = 51), with periclinal thickening and a small, flared collarette. Sporodochial macroconidia cylindrical to falcate, gently curved, typically with a blunt and almost rounded apical cell and a barely notched foot cell, 3–6-septate, hyaline, smooth, thin-walled. Three-septate conidia: (22.1–)22.6–39.4(–54.7) × (5.0–)5.5–6.7(–7.4) μm, (mean ± SD = 31.0 ± 8.4 × 6.1 ± 0.6 μm, n = 11); four-septate conidia: (50.3–)54.4–68.2(–69.6) × (6.9–)6.9–7.7(–8.0) μm, (mean ± SD = 61.3 ± 6.9 × 7.3 ± 0.4 μm, n = 10); five-septate conidia: (51.8–)60.6–73.0(–78.2) × (6.4–)6.1–7.1(–8.5) μm, (mean ± SD = 66.8 ± 6.2 × 6.6 ± 0.5 μm, n = 31); six-septate conidia: (69.8–)70.7–77.7(–79.6) × (7.1–)7.5–8.3(–8.3) μm, (mean ± SD = 74.2 ± 3.5 μm × 7.9 ± 0.4 μm, n = 10). Chlamydospores developed in large numbers in hyphae and also in mature macroconidia. The chlamydospores were 0–1-septate, globose to ellipsoidal, constricted at the septum, intercalary or terminal in chains or solitary with mostly a pale color and smooth, (11.7–)11.7–12.9(–13.5) × (7.7–)7.7–8.5(–8.6) μm, (mean ± SD = 12.3 ± 0.6 × 8.1 ± 0.4 μm, n = 6).

##### Culture characteristics.

Colonies on PDA growing in the dark with an average growth rate of 9.2 mm/d at 25 °C. Colony color white, flat, margins regular and fimbriate. Aerial mycelia abundant. Odor absent. Reverse white to pale luteous. Colonies on SNA incubated at 25 °C in the dark growing at 7.2 mm/d. Colony surface pure white, aerial mycelium scant. Reverse pure white, without diffusible pigments. Colonies on OMA incubated at 25 °C in the dark growing at 10.1 mm/d, color white, flat, velvety to felty with abundant floccose aerial mycelium. Reverse white without diffusible pigments. Colonies on CMA incubated at 25 °C in the dark were round, colony surface and reverse white, flat, aerial mycelium absent, hyphae hyaline, growing at 9.1 mm/d.

##### Additional materials examined.

China, Hunan province, Yiyang City, Heshan District, Henglongqiao Town, 28°27′24″N, 112°29′7″E, isolated from leaf spots of *Cunninghamialanceolata*, May 2017, Wen-Li Cui, isolates: HN33-8-2-1, HN33-8-2-2, HN33-8-2-3.

##### Notes.

The isolates of *F.hunanense* were phylogenetically close to *F.pseudensiforme* (ex-type, CBS 125729) (Fig. 4). Between *F.hunanense* isolates and ex-type of *F.pseudensiforme* CBS 125729, there were 8/583 differences in *TEF-1α*, 3/800 in *RPB2*, and 9/640 in *RPB1*. The PHI analysis showed that there was no significant recombination among *F.hunanense* isolates and its related species (Φ_w_ = 1.0) (Fig. 3B). Morphologically, 5-septate sporodochial macroconidia of the *F.hunanense* isolates (60.6–73.0 × 6.1–7.1 µm) were longer than those of *F.pseudensiforme* CBS 125729 (ex-type) (50–63 × 5.2–7.2 µm) (Nalim et al. 2011). In conclusion, the phylogenetic and morphological evidence supported this fungus being a new species within the *F.solani* species complex.

##### Pathogenicity assays.

**Figure 9.**
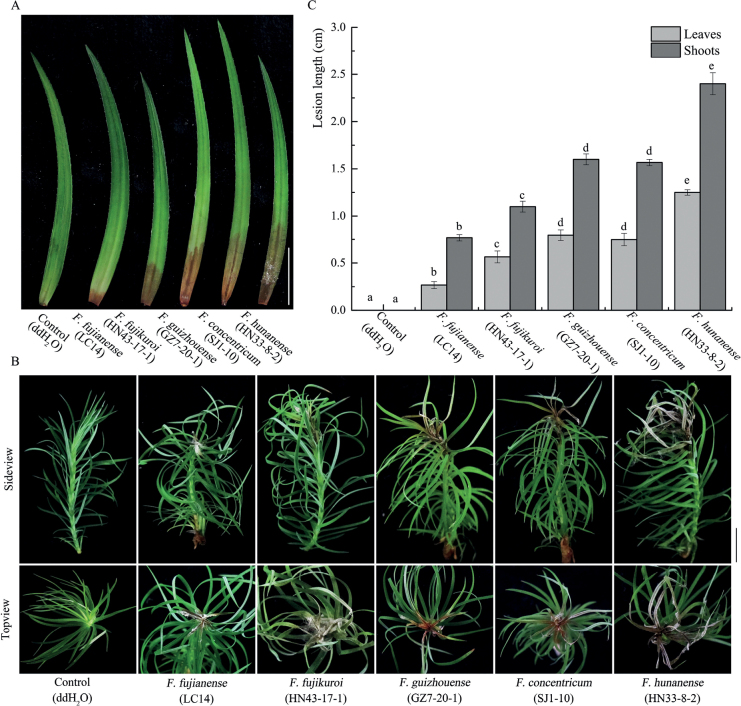
Symptoms on detached *Cunninghamialanceolata* leaves (**A**) and shoots of tissue-culture seedlings of *C.lanceolata* (**B**) inoculated with isolates: *Fusariumfujianense* (LC14), *F.fujikuroi* (HN43-17-1), *F.guizhouense* (GZ7-20-1), *F.concentricum* (SJ1-10), and *F.hunanense* (HN33-8-2). Scale bar: 10 mm. C, Lesion length on detached *C.lanceolata* leaves inoculated with *F.fujianense* (LC14), *F.fujikuroi* (HN43-17-1), *F.guizhouense* (GZ7-20-1), *F.concentricum* (SJ1-10), and *F.hunanense* (HN33-8-2). Error bars represent standard deviation, and different letters indicate significant difference based on LSD’s range test at *P* < 0.05 (n = 8).

Pathogenicity was tested on detached *C.lanceolata* leaves *in vitro* following Koch’s postulates for *F.hunanense* (HN33-8-2), *F.concentricum* (SJ1-10), *F.guizhouense* (GZ7-20-1), *F.fujikuroi* (HN43-17-1), and *F.fujianense* (LC14). At five days post-inoculation, all the tested isolates caused leaf necrosis, with dark brown lesions. The control remained unchanged (Fig. 9A). Equivalently, shoots of tissue-culture seedlings of *C.lanceolata* were inoculated by *F.hunanense* (HN33-8-2), *F.concentricum* (SJ1-10), *F.guizhouense* (GZ7-20-1), *F.fujikuroi* (HN43-17-1), and *F.fujianense* (LC14) *in vivo*. After ten days post-inoculation, all isolates caused necrotic lesions on shoots of *C.lanceolata*. The control remained healthy (Fig. 9B). Statistically, these isolates showed different levels of virulence. *Fusariumhunanense* (HN33-8-2) was significantly more virulent than those of *F.concentricum* (SJ1-10), *F.guizhouense* (GZ7-20-1), *F.fujikuroi* (HN43-17-1), and *F.fujianense* (LC14), while *F.fujianense* (LC14) was the least virulent (Fig. 9C).

The fungal isolates used for inoculation were re-isolated from the diseased spots on the inoculated leaves and shoots, but no fungus was isolated from the leaves and shoots of the control. Koch’s postulates were satisfied, and these isolates HN33-8-2, SJ1-10, GZ7-20-1, HN43-17-1, and LC14 were determined to be the pathogens of leaf blight on *C.lanceolata*.

## ﻿Discussion

In this study, the pathogens causing leaf blight of *C.lanceolata* in China, focusing especially on Fujian, Guangxi, Guizhou, and Hunan provinces, were determined by the inoculation tests using the shoots of tissue-culture seedlings of *C.lanceolata*. Phylogenetic and morphological analyses were used to evaluate the diversity of *Fusarium* species from the symptomatic *C.lanceolata* leaves. Three of the species newly described here (*F.fujianense*, *F.hunanense*, and *F.guizhouense*) and two known species (*F.fujikuroi* and *F.concentricum*) were associated with leaf blight of *C.lanceolata*. To date, F.oxysporumf.pini has been reported from *C.lanceolata* in Taiwan, China (Anonymous 1979). *Fusariumoxysporum* and *Fusarium* sp. have been reported to cause *C.lanceolata* seedlings damping off in mainland China (Chen 2002; Tian et al. 2019). However, none of the five species of *Fusarium* were previously reported to be pathogens of this disease. The taxonomic and phylogenetic analyses are the basis of research for various fields of *Fusarium* biology. Because often *Fusarium* isolates show morphological variation during their growth in culture, their identification faces certain difficulties and challenges. Microscopically, the most typical feature of the genus *Fusariums.l.* is its identifiable spindle- or canoe-shaped macroconidia (hyaline, multicellular, in clusters, macroconidia with or without foot cells at the base). If microconidia are present, the shape, number of cells, and mode of conidiogenesis (chains or false heads) are important in identification (Leslie and Summerell 2006).

Phylogenetic analyses based on DNA sequence diversity plays a crucial role, and many molecular markers, such as ITS, *TUB2*, *HIS3*, and *CAL* etc. have been used. However, *RPB2* and *TEF-1α* sequences appear to be the most useful in taxonomic studies of fungi, especially for the members of the genus *Fusarium* (O’Donnell 2000; O’Donnell et al. 2013; Crous et al. 2021). In the previous results of this study, it was found that, compared to *TEF-1α* and *RPB2* gene sequences, the ITS possesses relatively little phylogenetic signal, and the *TUB2* sequence is too short, thus the two loci have been eliminated. In the present study, the phylogeny inferred from concatenate multi-locus sequences (*TEF-1α*, *RPB2*, and *RPB1*) as suggested from previous studies (Sandoval-Denis et al. 2018) grouped isolates from *C.lanceolata* into five species belonging to four *Fusarium* species complexes with high supports. It should be noted here that, *TEF-1α*, *RPB2* and *RPB1* genes used to distinguish these species have rich information, but relatively few *RPB1* sequences are available in the databases, so there were some limitations using *RPB1*.

At present, the taxonomic studies on *Fusarium* are very divisive, especially segregating the *Fusariumsolani* species complex as *Neocosmospora* (Lombard et al. 2015; de Hoog et al. 2023). The disagreement has become wider in recent years. Both sides have their support. In addition to the previous publications, the studies published in 2023 reflect such a dilemma. Chen et al. (2023) recognized nine genera of fusarioid and considered these nine genera are well-supported in their present phylogenomic study and different from *Fusarium*, while Zeng and Zhuang (2023) recognized 14 genera. At the same time, some mycologists, plant pathologists, and medical mycologists supported the broad concept of *Fusarium* and preferred the species complexes of *Fusarium*. *Fusariumbilaiae* Gagkaeva & al., a new cryptic species from sunflower, has been described in the *Fusariumfujikuroi* species complex using the tef, tub, and rpb2 sequences (Gagkaeva et al. 2023). In a Brazilian study on *Fusarium* from melons, Silva et al. (2023) favored *Fusariumsolani* species complex (FSSC) and reported that among the 31 isolates, 29 isolates were *Fusariumfalciforme* (Carrión) Summerb. & Schroers, (=*Neocosmosporafalciformis* (Carrión) L. Lombard & Crous) and two isolates were *F.suttonianum* (Sand.-Den. & Crous) O’Donnell, Geiser & T. Aoki (≡*Neocosmosporasuttoniana* Sand.-Den. & Crous) using sequences of *EF-1α* and *RPB2*. The position paper by de Hoog et al. (2023) to the medical community showed how complicated the disagreement has become at present. de Hoog et al. (2023) indicated that the phylogenetic relationship between *Fusarium* and *Neocosmospora* may justify their segregation, and it seems necessary to maintain the fusarium-like genera proposed by Crous et al. (2021). However, de Hoog et al. (2023) also opined that the segregation of *Neocosmospora* was not obligatory for the medical fields to be adopted immediately and recommended waiting until taxonomists settle their disagreement (de Hoog et al. 2023). Thus, de Hoog et al. (2023) recommended using the names under *Fusarium* species complexes, not the names under the segregated genera. This is the opinion with which we agree.

Species delineation needs polyphasic support. In addition to phylogenetic analyses and morphological studies, genealogical concordance analysis enables to determine sexual recombination and provides an operational criterion to verify the species borderline (de Hoog et al. 2023). This method was used in our present studies and no significant genetic recombination was in the new species that we described.

Pathogenicity tests showed that all five species were able to infect host plants. However, these species displayed differences in virulence on *C.lanceolata*. It is well known that *F.fujikuroi* is the causal agent of the rice disease bakanae in the major rice-growing regions in the world (Leslie and Summerell 2006). Besides rice, *F.fujikuroi* has been reported as saprobe or endophyte of vanilla (Pinaria et al. 2010) and isolated from human skin (O’Donnell et al. 2010). However, the predominant presence of *F.fujikuroi* from leaves of *C.lanceolata* has not been reported. This result could also be explained by the crop planting history of the sample site. We speculated that the fields have been previously planted with rice, which are highly susceptible to *F.fujikuroi* among other *Fusarium* species. *Fusariumconcentricum* was described as a new species by Nirenberg and O’Donnell (1998), which was predominantly isolated from Musa×paradisiaca (banana) in Central America and *Nilaparvatalugens* (Asian brown leaf hopper) in South Korea. *Nilaparvatalugens* is a serious pest on rice in Asia (Wu et al. 2018). It is possible that this insect serves as a vector for this pathogen’s dispersal. Very little is known about the pathogenicity and biology of *F.concentricum* (Leslie and Summerell 2006). However, *F.fujikuroi* and *F.concentricum* are reported to cause leaf blight on *C.lanceolata* for the first time.

The present study introduces new insights into the biodiversity of *Fusarium* species associated with *C.lanceolata* in China. A remarkable diversity of *Fusarium* species spanning several species complexes was found from four provinces, China. Furthermore, three new species of *Fusarium* were described, with demonstrated pathogenicity to *C.lanceolata*. However, considering the limited geographic areas studied, it is likely that additional *Fusarium* species would also be isolated if more areas were studied. Meanwhile, this also shows that despite the widespread distribution of *C.lanceolata* in China, and previous knowledge about its associated microbes, the fungal species-richness in *C.lanceolata* remains underestimated. Therefore, more studies are necessary on these new taxa in order to elucidate their host range, specificity, and global distribution, as well as their potential impact on the *C.lanceolata* industry.

## Supplementary Material

XML Treatment for Fusarium concentricum

XML Treatment for Fusarium fujikuroi

XML Treatment for Fusarium fujianense

XML Treatment for Fusarium guizhouense

XML Treatment for Fusarium hunanense

## ﻿Additional information

### Author ORCIDs

Jiao He https://orcid.org/0000-0002-4146-2223

De-Wei Li https://orcid.org/0000-0002-2788-7938

Wen-Li Cui https://orcid.org/0009-0005-7515-7672

Li-Hua Zhu https://orcid.org/0000-0003-2740-4980

Lin Huang https://orcid.org/0000-0001-7536-0914

### Data availability

All of the data that support the findings of this study are available in the main text or Supplementary Information.

## Supplementary materials

Supplementary material 1 Supplementary data Data type docx Explanation note **table S1.** Fungal cultures isolated from Chinese fir in this study. **table S2.** Genes/region and respective primer pairs used in the study. **table S3.** Nucleotide substitution models used in the phylogenetic analyses. **fig. S1.***Fusariumconcentricum* (SJ1-10). A–D, Colonies on PDA, SNA, OMA, and CMA, respectively, after 5 days at 24°C in the dark; E–F, sporodochia formed on PDA and the surface of carnation leaves, respectively; G–H, aerial conidiophores; I–J, sporodochial conidiophores, phialides, and conidia; K–L, aerial phialides and conidia; M, microconidia (0–1-septate) and macroconidia (3–5-septate). **fig. S2.***Fusariumfujikuroi* (HN43-17-1). A–D, Colonies on PDA, SNA, OMA, and CMA, respectively, after 5 days at 24°C in the dark; E–H, aerial conidiophores, phialides, and microconidia; H, microconidia (0-septate); I, chlamydospore. https://binary.pensoft.net/file/963607This dataset is made available under the Open Database License (http://opendatacommons.org/licenses/odbl/1.0/). The Open Database License (ODbL) is a license agreement intended to allow users to freely share, modify, and use this Dataset while maintaining this same freedom for others, provided that the original source and author(s) are credited.Jiao He, De-Wei Li, Wen-Li Cui, Li-Hua Zhu, Lin Huang
