# Supplementary material for: ﻿Morphological and phylogenetic analyses reveal three new species of Fusarium (Hypocreales, Nectriaceae) associated with leaf blight on Cunninghamialanceolata in China
Source: MycoKeys. 2024 Jan 8;101:45–80. doi: 10.3897/mycokeys.101.113128 (PMC10790579; doi:10.3897/mycokeys.101.113128)

**Supplementary Materials**

**Supplementary Table 1. Fungal cultures isolated from Chinese fir in this study**

| Strian code | Location | Longitude | Latitude |
| --- | --- | --- | --- |
| LC14 | Yangkou Forest Farm, Shunchang County, Nanping City, Fujian Province, China | 117°52'48" | 26°48'36" |
| LC14-1 |  |  |  |
| LC14-2 |  |  |  |
| LC14-3 |  |  |  |
| GZ7-20-1 | Kelou Town, Cengong County, Qiandongnan Miao and Dong Autonomous Prefecture, Guizhou province, China | 108°22'9" | 27°22'58" |
| GZ7-20-1-1 |  |  |  |
| GZ7-20-1-2 |  |  |  |
| GZ7-20-1-3 |  |  |  |
| HN33-8 | Henglongqiao Town, Heshan District, Yiyang City, Hunan Province, China | 112°29'7" | 28°27'24" |
| HN33-8-1 |  |  |  |
| HN33-8-2 |  |  |  |
| HN33-8-3 |  |  |  |
| HN43-17 |  |  |  |
| HN43-17-1 |  |  |  |
| HN43-17-2 |  |  |  |
| HN43-17-3 |  |  |  |
| SJ1-10 | Guyi Town, Sanjiang Dong Autonomous County, Liuzhou City, Guangxi Zhuang Autonomous Region | 109°28'47" | 25°25'48" |
| SJ1-10-1 |  |  |  |
| SJ1-10-2 |  |  |  |
| SJ1-10-3 |  |  |  |

**Supplementary Table 2.** Genes/region and respective primer pairs used in the study.

| Genes/regions | Primer name | Sequence^a^ |
| --- | --- | --- |
| Translation elongation  factor 1-alpha (*TEF-1α*) | EF1 | 5’- ATG GGT AAG GAG GAC AAG AC -3’ |
|  | EF2 | 5’-GGA GGT ACC AGT GAT CAT G -3’ |
| RNA polymerase second  largest subunit (*RPB2*) | 5f2 | 5’- GGG GTG ATC AGA AGA AGG C -3’ |
|  | 7cr | 5’- CCC ATG GCT TGT TTG CCC AT -3’ |
| RNA polymerase  largest subunit (*RPB1*) | Fa | 5’- CAY AAR GAR TCY ATG ATG GGW C -3’ |
|  | G2R | 5’- GTC ATY TGD GTD GCD GGY TCD CC -3’ |
| a D = A, G or T; R = A or G; W = A or T; Y = C or T. | | |

**Supplementary Table 3.** Nucleotide substitution models used in the phylogenetic analyses.

| Species complex | Locus^1^ | Length of genes/regions (including gap) | Nucleotide substitution models^2^ | |  |
| --- | --- | --- | --- | --- | --- |
|  |  |  | ML | BI |  |
| *Fusarium fujikuroi* species complex | RPB1 | 901 | AIC: GTR+F+G4 | BIC: K2P+G4 |  |
|  | RPB2 | 791 | AIC: GTR+F+I+G4 | BIC: K2P+G4 |  |
|  | TEF-1α | 527 | AIC: GTR+F+G4 | BIC: SYM+G4 |  |
|  | combined | 2219 | AIC: GTR+F+I+G4 | BIC: SYM+I+G4 |  |
| *F. lateritium* species complex | RPB1 | 615 | AIC: GTR+F | BIC: SYM |  |
|  | RPB2 | 776 | AIC: GTR+F+G4 | BIC: SYM+G4 |  |
|  | TEF-1α | 672 | AIC: GTR+F+I | BIC: K2P+I |  |
|  | combined | 2063 | AIC: GTR+F+G4 | BIC: SYM+I |  |
| *F. solani* species complex | RPB1 | 640 | AIC: GTR+F+I+G4 | BIC: SYM+G4 |  |
|  | RPB2 | 800 | AIC: GTR+F+G4 | BIC: SYM+G4 |  |
|  | TEF-1α | 583 | AIC: GTR+F+I+G4 | BIC: SYM+G4 |  |
|  | combined | 2023 | AIC: GTR+F+I+G4 | BIC: SYM+I+G4 |  |
| *F. sambucinum* species complex | RPB1 | 641 | AIC: GTR+F+I+G4 | BIC: SYM+G4 |  |
|  | RPB2 | 897 | AIC: GTR+F+G4 | BIC: SYM+G4 |  |
|  | TEF-1α | 577 | AIC: GTR+F+G4 | BIC: SYM+G4 |  |
|  | combined | 2115 | AIC: GTR+F+I+G4 | BIC: SYM+G4 |  |
| 1 TEF-1α: Translation elongation factor 1-alpha; RPB2: RNA polymerase second largest subunit; RPB1: RNA polymerase largest subunit. 2 G: Gamma distributed rate variation among sites; GTR: Generalized time-reversible; I: Proportion of invariable sites; K2P: Kimura 2-Parameter; SYM: Symmetrical model; F: Felsenstein. | | | | |  |
|  |  |  |  |  |  |

**Supplementary Figures**

**Figure S1.** *Fusarium concentrium* (SJ1-10). A–D, Colonies on PDA, SNA, OMA, and CMA, respectively, after 5 days at 24°C in the dark; E–F, sporodochia formed on PDA and the surface of carnation leaves, respectively; G–H, aerial conidiophores; I–J, sporodochial conidiophores, phialides, and conidia; K–L, aerial phialides and conidia; M, microconidia (0–1-septate) and macroconidia (3–5-septate). Scale bars: E–F=500 μm; G–H = 50 μm; I–M = 10 μm.


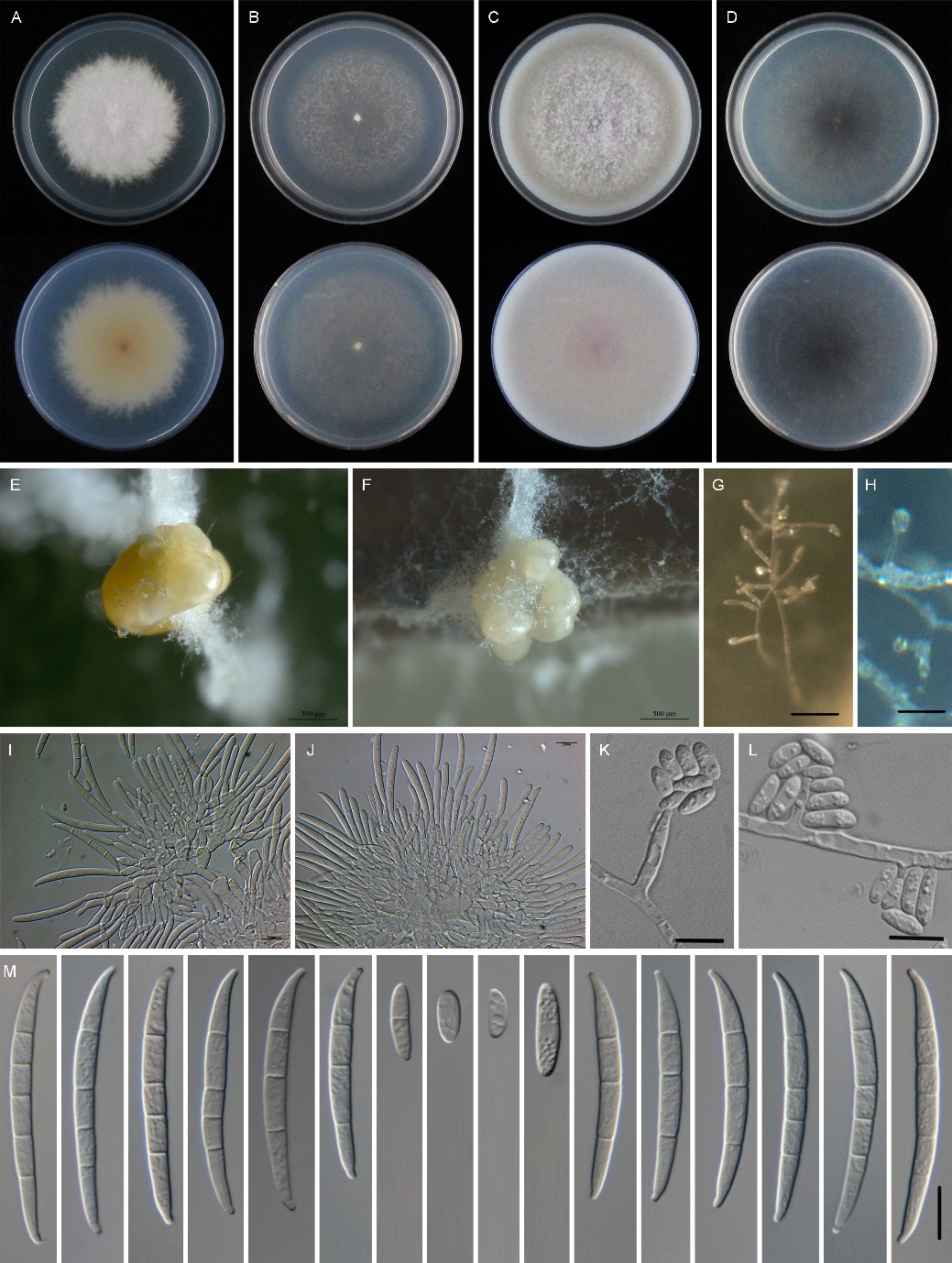


**Figure S2.** *Fusarium fujikuroi* (HN43-17-1). A–D, Colonies on PDA, SNA, OMA, and CMA, respectively, after 5 days at 24°C in the dark; E–H, aerial conidiophores, phialides, and microconidia; H, microconidia (0-septate); I, chlamydospore. Scale bars: E = 50 μm; F–I = 10 μm.


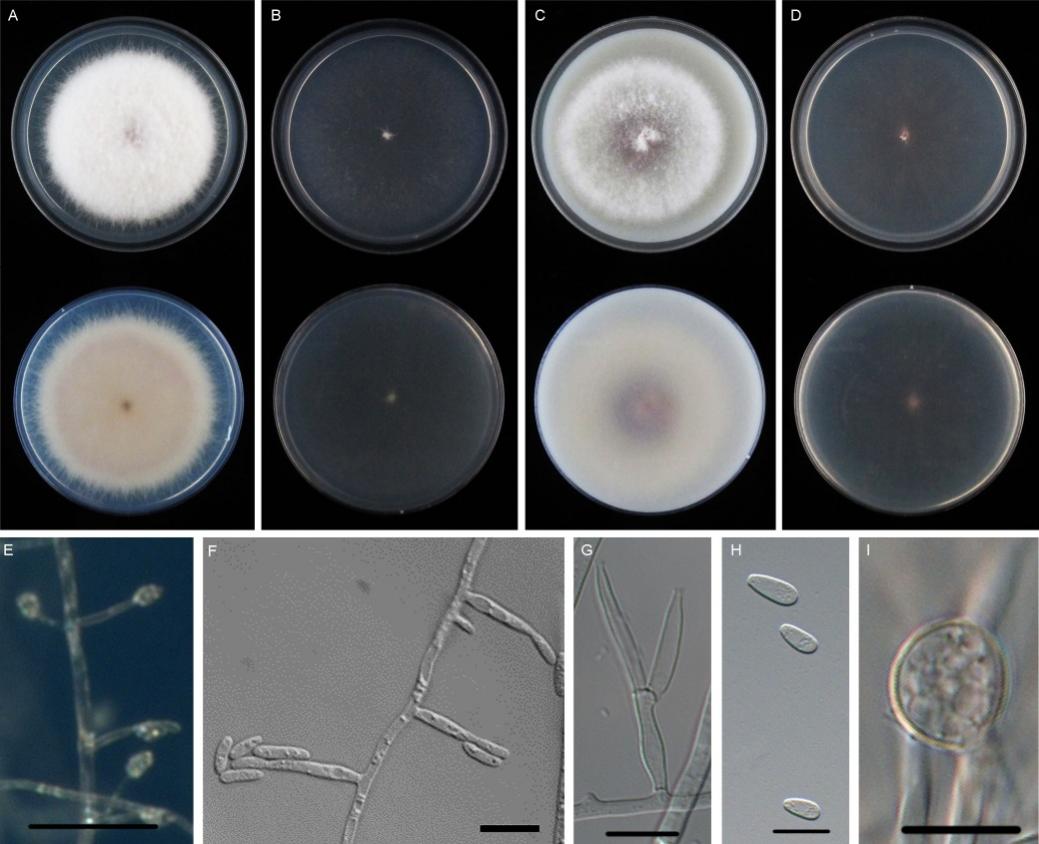

Supplement: Supplementary material 1 — Supplementary data [file mycokeys-101-045-s001.docx]
